# Physical determinants of reliable RNA–ligand binding prediction revealed by P-RISE

**DOI:** 10.1093/nar/gkag1062

**Published:** 2026-11-02

**Authors:** Hui Li, Youchao Deng, Zhuoxi Jiang, Xinyu Gu, Dejun Jiang, Dongsheng Cao

**Affiliations:** Xiangya School of Pharmaceutical Sciences, Central South University, Changsha 410004, China; Xiangya School of Pharmaceutical Sciences, Central South University, Changsha 410004, China; Xiangya School of Pharmaceutical Sciences, Central South University, Changsha 410004, China; Xiangya School of Pharmaceutical Sciences, Central South University, Changsha 410004, China; Xiangya School of Pharmaceutical Sciences, Central South University, Changsha 410004, China; Xiangya School of Pharmaceutical Sciences, Central South University, Changsha 410004, China

## Abstract

RNA-targeted small-molecule discovery is challenged by the scarcity of experimentally characterized RNA–ligand complexes and by the limited transferability of current computational approaches across chemically diverse systems. Here we present P-RISE, a physics-grounded framework for RNA–ligand binding prediction, and use it to identify the physical determinants underlying reliable performance. Systematic evaluation of force fields, ionic environments, dielectric treatments, and entropy corrections reveals three coupled requirements: preservation of RNA-binding pocket integrity under ion-sensitive conditions; an effective electrostatic error cancellation regime enabled by an elevated internal dielectric constant; and an empirically observed coupling between entropy correction and solvent modeling. Integrating these features, P-RISE provides stable affinity ranking, physics-grounded delineation of its physicochemical applicability domain, accurate metal ion site localization, and improved binding pose discrimination relative to established RNA docking and scoring approaches. Crucially, benchmarking against AlphaFold3 reveals that co-folding models, optimized for structure generation rather than energetic ranking, benefit from an explicit physics-based evaluation layer. Furthermore, diverse machine-learning comparisons demonstrate that purely data-driven methods lack stability and generalization in this data-poor regime. Ultimately, P-RISE provides a mechanistic understanding of RNA–ligand modeling, serving as a principled physical baseline that defines reliable prediction boundaries, and guides future data-driven integration.

## Graphical Abstract

**Graphical Abstract:**
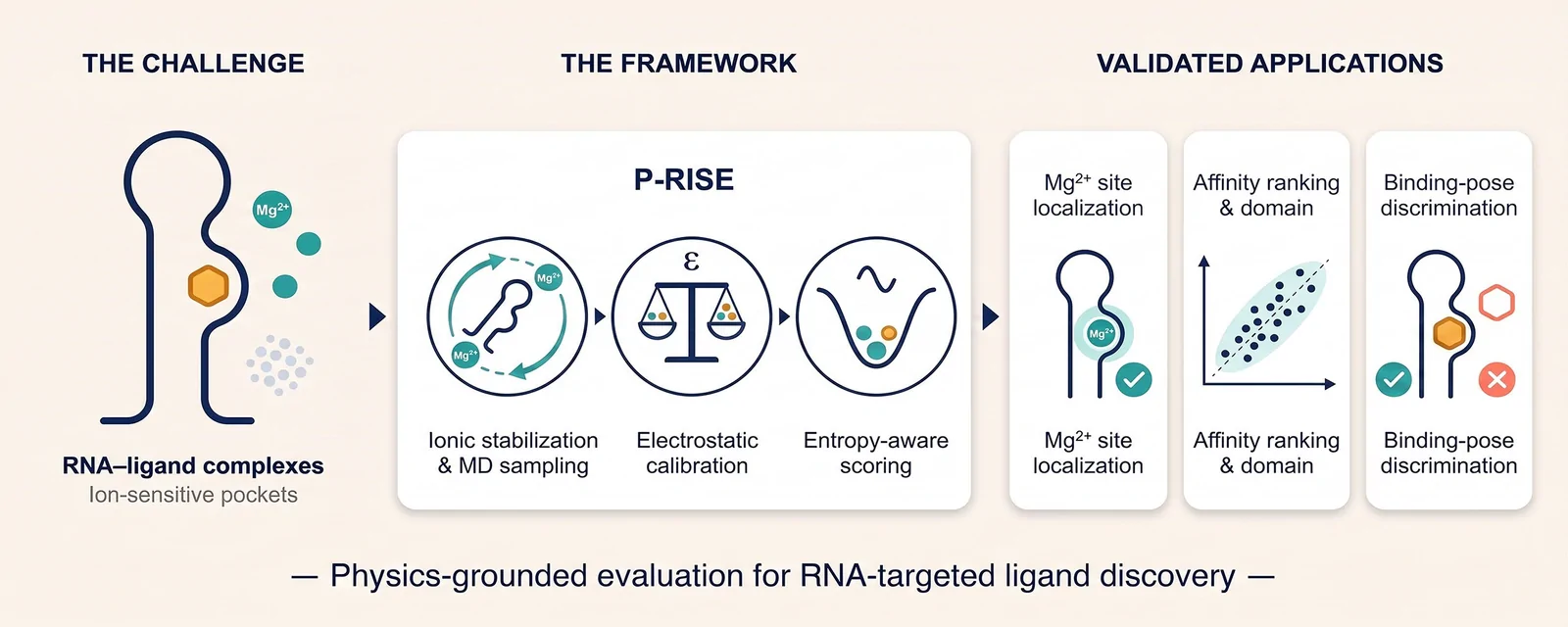

## Introduction

RNA has emerged as a major class of drug targets due to its diverse functional and regulatory roles in gene expression, protein synthesis, and viral replication. As a central component of molecular biology’s central dogma, RNA plays a critical role in the regulation and expression of genetic information, making it a highly attractive target for molecular medicine [1]. The identification of compounds that specifically bind to RNA targets represents a promising therapeutic strategy for diseases associated with RNA dysfunction. Compared with protein-targeted approaches, targeting RNA significantly broadens the druggable fraction of the genome and offers the potential for more effective disease intervention by leveraging the pivotal roles of RNA. It is estimated that ~85% of the human genome is transcribed into RNA, while only ~1% encodes proteins, and only a small fraction of these proteins are viable drug targets. The vast majority of transcribed RNAs are non-coding RNAs (ncRNAs), which perform essential enzymatic, structural, and regulatory roles instead of encoding proteins [2]. Recent studies have revealed that many ncRNAs, including microRNAs (miRNAs), long non-coding RNAs (lncRNAs), and circular RNAs (circRNAs), are implicated in human diseases such as cancer and neurodegenerative disorders, positioning them as promising new drug targets [3]. The discovery that ribocil and its derivatives can bind to the flavin mononucleotide (FMN) riboswitch to inhibit riboflavin synthesis demonstrated the feasibility of targeting RNA with small molecules, underscoring the urgent need for efficient and reliable methods to predict RNA–ligand binding affinities [4].

The growing interest in RNA-targeted drug discovery has driven the maturation of experimental and computational methods for delineating RNA–small molecule interactions. Experimentally, biophysical techniques such as nuclear magnetic resonance (NMR), isothermal titration calorimetry (ITC), and X-ray crystallography have seen significant progress. For instance, recent breakthroughs such asscaffold-enabled cryogenic electron microscopy (cryo-EM) are pushing the boundaries of structural resolution for challenging RNA targets [5], while high-throughput techniques such as fluorescence resonance energy transfer (FRET)-based conformational mapping now enable the detailed quantification of how factors such as Mg^2+^ concentration modulate RNA stability and affinity [6]. Together, these advances have made the direct determination of RNA–small molecule binding modes and affinities increasingly achievable, providing a solid foundation for the development and validation of theoretical models.

Computationally, approaches for identifying lead compounds for specific RNA targets can be broadly categorized into data-driven methods and physics-based structural methods. Data-driven methods (e.g. QSAR and deep learning models) have been applied across multiple stages of RNA drug discovery. For instance, in the target discovery and pocket identification stage, tools such as fpocketR [7], DRLiPS [8], DrugPred_RNA [9], MultiModRLBP [10], and RPflex [11] are employed to identify and characterize druggable binding pockets on RNA structures. In the subsequent lead discovery phase, methods such as fingeRNAt [12], the sequence-based deep learning approach RNAsmol [13], and the deep graph learning-based RNAmigos2 [14] facilitate high-throughput virtual screening of vast compound libraries to enrich potential active molecules. In the binding pose generation stage, co-folding models represented by AlphaFold3 [15] have begun to be used to generate RNA–ligand complex structures. In contrast to the end-to-end prediction paradigm partially embodied by these data-driven co-folding models, in traditional workflows, binding pose prediction and pose evaluation are treated as two distinct and explicitly separated computational tasks. The former focuses on generating possible binding modes within the conformational space, while the latter concerns identifying the pose closest to the native state from these candidates. These tasks have long relied on empirical or physical approximation methods and are typically concatenated into a “docking generation + scoring screening” workflow. In the pose prediction stage, specialized docking programs such as RLDOCK [16, 17], riboDock [18], its successor rDock [19], NLDock [20], and PLANTS [21] generate candidate binding modes through conformational search algorithms and simplified energy functions. Subsequently, in the pose evaluation stage, specialized scoring functions such as RmsdXNA [22] and AnnapurnaRNA [23] are utilized to assess the plausibility of candidate ligand binding poses. While these methods have been widely applied in protein–ligand systems, their reliability and applicability boundaries in highly flexible RNA systems lack systematic assessment. In stark contrast, physics-based methods approach the problem from a different theoretical standpoint, focusing on quantifying RNA–ligand interactions by calculating relative or absolute binding free energy. For example, Dejun Jiang *et al*. conducted a detailed assessment of endpoint free energy methods for RNA–small molecule systems [24]. Ali Rasouli and colleagues utilized BFEE2 [25] to investigate the absolute binding free energies of RNA with six theophylline analogs [26]. More recently, Schrödinger, Inc. extended its free energy peturbation (FEP+) methodology to accommodate DNA/RNA–ligand systems [27]. These methods provide vital computational tools for understanding RNA–ligand binding.

Despite the significant progress made by the aforementioned methods in RNA–ligand research, the field still faces a series of persistent challenges. The inherent flexibility of RNA makes the experimental determination of its structures, particularly in complex with ligands, exceedingly difficult [28]. This leads to a severe scarcity of high-resolution experimental data. To illustrate the magnitude of this data disparity, as of 2025, the renowned PDBbind database contains >27 000 protein–ligand complexes but merely ∼100 RNA–ligand structures, which is a difference of nearly two orders of magnitude. This vast data gap constitutes a major bottleneck for training data-driven models. In this typical “data-poor regime”, the generalization ability of artificial intelligence (AI) models is markedly limited in practice, leading to substantial statistical instability in high-dimensional tasks such as conformational discrimination and precise energy ranking [29–31].

Against this background, computational methods grounded in physical first principles, such as free energy calculations, are widely considered a more reliable choice at the current stage. However, the exploration of physics-based methods for RNA–ligand systems is still in its infancy, and their predictive accuracy urgently needs improvement. While alchemical methods such as Schrödinger’s FEP+ are widely regarded as having a rigorous physical basis and leading performance in predicting RNA–ligand binding affinities, they are designed to compute relative rather than absolute binding free energies. Furthermore, although studies indicate the necessity of ions for structural stabilization, the field lacks standardized guidance on specific ion placement strategies and equilibration protocols for simulation systems. Moreover, the prohibitive computational cost of these methods limits their widespread application in early-stage drug screening [27]. As an efficient alternative, endpoint methods such as the Molecular Mechanics/Generalized Born Surface Area (MM/GBSA) are widely used, but their application to RNA–ligand systems has largely remained in an empirical, “trial-and-error” stage. Our own preliminary research underscored this limitation, showing that simply fine-tuning conventional parameters yielded only modest predictive accuracy (*R*_p_ = 0.513) [24]. This suboptimal performance reflects a lack of systematic understanding of the key physicochemical determinants governing predictive reliability in RNA systems. How can one construct a stable and realistic simulation system (including ionic environment settings) for highly flexible RNA? How do factors such as solvent models, dielectric constants, and entropy corrections compensate for the intrinsic deficiencies of the underlying force field? What are the theoretical applicability boundaries of endpoint methods? Owing to these fundamental issues, particularly the unresolved question of which physical factors determine the accuracy of RNA–ligand binding predictions, existing studies often resort to parameter guessing rather than establishing a robust and trustworthy computational protocol based on physical intuition.

Here, we present P-RISE, a physics-grounded framework for RNA–ligand binding prediction, and use it to systematically dissect the physical factors that determine predictive reliability across diverse RNA systems. Rather than focusing on a single optimized parameter set, we examine how force fields, ionic environments, dielectric treatments, and entropy corrections jointly shape the stability and transferability of RNA–ligand free energy estimation. This analysis identifies three coupled determinants of reliable prediction: preservation of RNA-binding pocket integrity under ion-sensitive conditions; an effective electrostatic regime enabled by an elevated internal dielectric constant; and an empirically observed coupling between the entropy correction and the choice of solvent model that improves cross-system predictive performance.

We integrate these principles into a unified framework (Fig. 1) and evaluate its performance across multiple RNA-relevant tasks, including affinity ranking, systematic validation of its physicochemical applicability domain boundaries, metal-ion site localization, and binding pose discrimination. Furthermore, by extensively benchmarking P-RISE against cutting-edge AI predictors, ranging from AlphaFold3 to hybrid machine-learning (ML) models, we characterize how closely current data-driven models can approach a physics-based reference under present RNA data constraints. We demonstrate that explicit physical modeling provides structural sensitivity and energetic reliability that complement current data-driven methods when training data are severely limited. Ultimately, this work not only establishes a highly interpretable and transferable strategy for practical RNA–ligand prediction, but also serves as a vital physical ground truth to critically evaluate and guide the future development of AI in RNA-targeted drug discovery.

**Figure 1.**
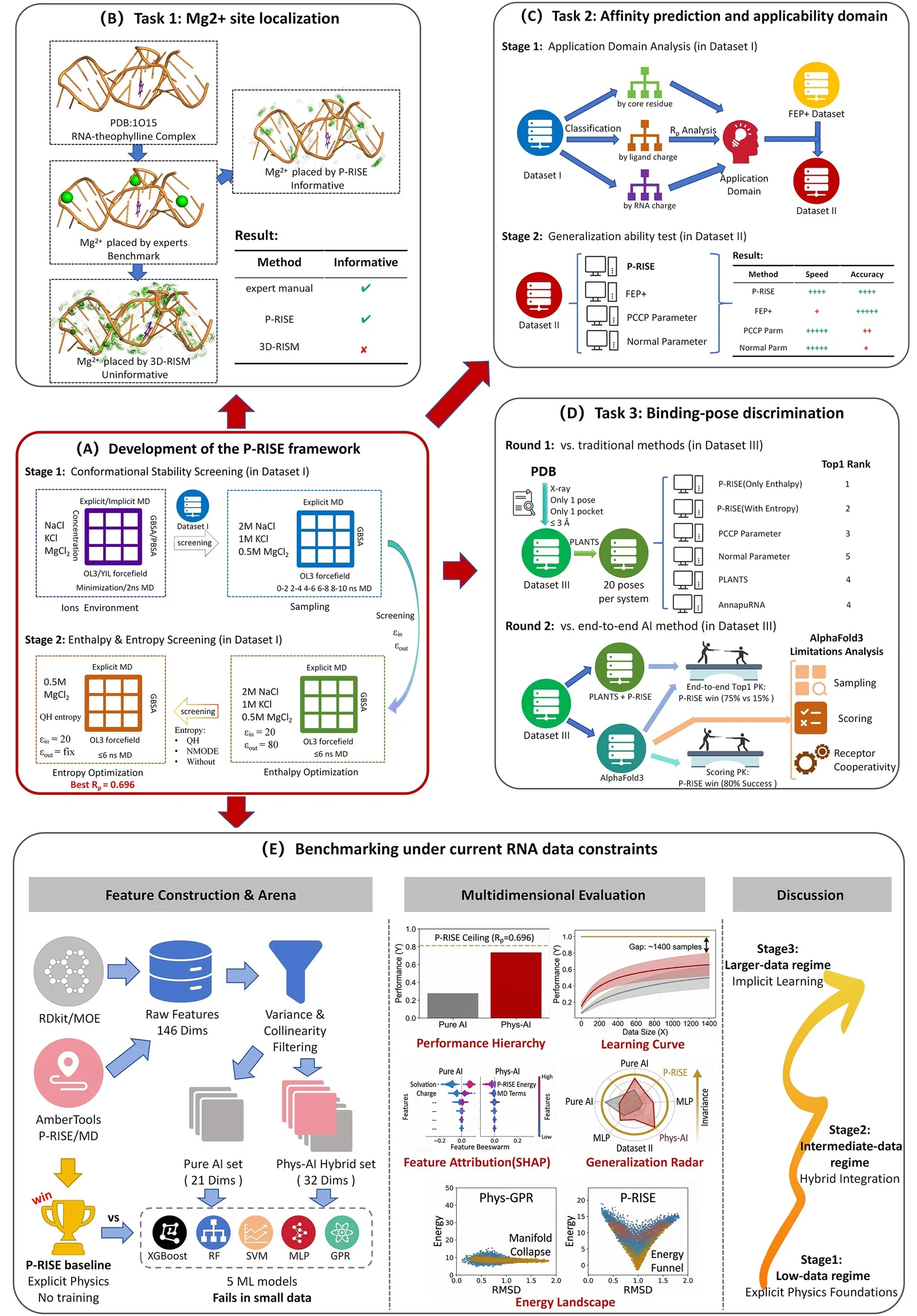
Overview of the P-RISE framework and its multi-dimensional validation. (**A**) Development of the P-RISE framework: two-stage parameter screening on Dataset I, spanning the ionic environment, sampling windows, entropy treatment, and dielectric settings. (**B)** Task 1: Mg^2+^ site localization, benchmarked against expert-assigned and 3D-RISM placements. (**C**) Task 2: affinity prediction and applicability domain analysis on Datasets I and II. (**D**) Task 3: binding pose discrimination on Dataset III against traditional scoring methods and end-to-end AI methods. (**E**) Benchmarking of descriptor-only and physics-augmented ML models under current RNA data constraints.

## Materials and methods

### Dataset assembly and preparation

Three distinct datasets were employed in this study to develop and validate our methodology.

#### Dataset I (primary affinity set)

This set was compiled for the initial exploration and optimization of parameters for binding affinity prediction. The set was adapted from a prior study in *Physical Chemistry Chemical Physics* [24] and initially consisted of 29 RNA–ligand complexes with high-resolution crystal structures (≤3.0 Å) and experimentally measured binding affinities. Following a structural integrity check (Supplementary Fig. S1; Supplementary Tables S1 and S2), two systems containing asymmetric dual ligands (PDB IDs: 4LVW and 4LVY) were excluded. This exclusion was performed to prevent potential ambiguities in the free energy calculations.

#### Dataset II (external free energy validation set)

An external validation set was curated to assess the generalizability of P-RISE for binding free energy prediction. This set was selected based on systems reported in a previous RNA–ligand FEP study [27]. Dataset construction was guided by the application domain framework defined in this work, which delineates the physicochemical conditions under which reliable predictions are expected. The boundaries of this domain are primarily demarcated by the following physicochemical characteristics: (i) the overall charge and structural category of RNA targets; (ii) the spectrum of charge states of ligands; and (iii) the type of energy contributions from residues that assume a dominant role in the binding mode. Based on this framework, we have selected two subsets (Benzimidazole and Rbt) (Supplementary Tables S3 and S4). In accordance with our application domain analysis, the physicochemical properties of these two systems fall entirely within the high-precision prediction application domain established in this study. Consequently, they serve as within-domain reproducibility sets: because the domain boundaries were fixed on Dataset I prior to and independently of the selection of these systems, they test whether the P-RISE protocol reproduces high accuracy on chemotypes that are structurally distinct from Dataset I but fall within the pre-defined domain. We emphasize that these subsets are not intended to demonstrate generalization beyond the domain; that claim rests exclusively on the out-of-domain Flavins subset described below. To probe the robustness of the defined application domain boundaries, an additional subset (Flavins; Supplementary Table S5) was included as an out-of-domain test case. In this system, the charge of the ligand transitions from −1 to 0, extending beyond both the calibrated prediction domain and the charge range represented in Dataset I. This thereby subjects the model’s robustness to a more rigorous scrutiny. Notably, datasets designed for relative FEP calculations often feature ligands with minimal structural and activity variations. We therefore selectively curated a subset of these ligands. This curation process ensured a sufficient dynamic range in binding affinities, a critical factor for robust correlation analysis.

#### Dataset III (pose prediction benchmark set)

A novel benchmark set was constructed specifically to evaluate binding pose prediction accuracy. First, all available RNA–ligand co-crystal structures were retrieved from the Protein Data Bank (PDB). To ensure high data quality, we applied several stringent filtering criteria. Only X-ray structures with a resolution better than 3.0 Å were included. Additionally, complexes were required to contain a single, non-covalently bound ligand. All NMR-derived structures were excluded from this set because a single PDB entry typically contains multiple NMR conformers. Crucially, the validity of a pose prediction benchmark depends on the availability of a single, unambiguously defined native reference structure for root mean square deviation (RMSD)-based evaluation. Our stringent filtering process, which prioritizes this structural fidelity, is therefore an essential prerequisite for constructing a high-quality and challenging evaluation. This filtering process yielded 20 unique complex systems. Subsequently, we generated a diverse set of decoy poses for each complex. This procedure was adapted from the decoy generation strategy used in the CASF benchmark for protein–ligand interactions [32–34]. To ensure that the generated decoy poses were both high in quality and sufficiently challenging, the choice of docking software was critical. Based on a previous systematic benchmarking study published in the *Journal of Chemical Theory* and *Computatio*n [35], which demonstrated that PLANTS exhibits the best overall performance among multiple docking tools for RNA–ligand systems, we selected PLANTS as the sole pose generation tool [21, 35]. This choice ensures that the benchmark is constructed using state-of-the-art docking performance, thereby providing a stringent evaluation of scoring function discrimination. For each RNA–ligand system, 100 binding poses were generated using PLANTS. The remaining decoys were categorized into 10 groups based on their RMSD to the native pose in 1 Å increments. Two poses were then randomly selected from each 1 Å bin for inclusion in Dataset III. In a limited number of systems, when PLANTS did not achieve sufficient sampling, we supplemented near-native conformations (RMSD <3 Å) by imposing minor random translations and rotations on the crystal ligands. For intervals within the range of 3–10 Å that were undersampled, we randomly selected from the pool of all generated docking conformations within this interval to complete the sampling. In line with the 3.0 Å resolution criterion adopted for screening crystal structures, we designated an RMSD of <3.0 Å as the pivotal criterion for assessing the success of conformation prediction. Supplementary Fig. S2 vividly showcases the diversity of the poses we selected. In this figure, the ordinate represents the RMSD between the ligand and the crystal ligand, the abscissa corresponds to the 20 PDB systems under our study, and the percentage numbers at the bottom denote the percentage of ligand poses with an RMSD <3 Å among all poses.

To provide a knowledge-based statistical reference, AnnapuRNA [23] was included as a representative control model. AnnapuRNA is a widely used scoring function for RNA–ligand interactions, derived from statistical potentials of experimentally resolved structures. All decoy conformations from Dataset III were evaluated using AnnapuRNA following its default protocol, and the resulting scores were used for pose ranking.

AlphaFold3 structure predictions were performed for the same 20 RNA–ligand complexes. We carried out structure predictions using random seeds from 1 to 20 separately. For each random seed, five complex conformations were generated, resulting in a total of 100 distinct complex conformations. The predicted complex structures were subsequently ranked for each system. We employed the US-align [36] tool to align the RNA of the crystal structure with the RNA predicted by AlphaFold3, thereby obtaining the TM-score. The RMSD was computed by comparing the ligand of the crystal structure with the predicted ligand subsequent to the RNA structure alignment performed by US-align. We note that the exclusion of NMR entries is a technical requirement of the single-reference RMSD metric and applies to Dataset III only; it does not imply that NMR structures are less informative. Conformational flexibility and dynamic ensembles of RNA are represented in this work through explicit molecular dynamics (MD) sampling performed for every system rather than through experimentally deposited conformer sets. As an independent test of this design choice, an ensemble-aware control set based on NMR structures was additionally constructed.

#### Ensemble-aware NMR control set

To assess the potential bias introduced by restricting Dataset III to crystallographic structures, 41 NMR-derived RNA–ligand complexes containing an explicitly, non-covalently bound ligand were retrieved from the PDB. For each entry, all deposited conformers were first superimposed onto a common frame using RNA heavy atoms; PLANTS docking was then performed independently against every conformer, and the resulting poses from all conformers were pooled into a single pose set (ensemble docking). A controlled decoy set was subsequently constructed following exactly the protocol used for Dataset III: two poses were randomly selected from each 1 Å bin spanning 0–10 Å, and undersampled near-native bins (RMSD <3 Å) were supplemented by imposing minor random translations and rotations on the deposited ligands. The final control set comprises 797 poses, of which 26.5% are near-native (RMSD <3 Å), matching the proportion in the crystallographic set; all 797 poses were subjected to the same MD equilibration and P-RISE protocol used throughout this work. Ligand RMSD was computed with an algorithm that automatically resolves atom correspondence and topological symmetry equivalence. Because an NMR entry provides multiple equally valid deposited conformers, two evaluation criteria were defined: the best-match RMSD, taken as the minimum ligand RMSD of a pose over all *N* deposited conformers of the entry; and the ensemble-average RMSD, taken as the arithmetic mean over the same *N* conformers. The ensemble divergence of a system was defined as the mean pairwise RMSD between ligand heavy atoms across the superimposed conformers, quantifying the intrinsic dispersion of the experimental reference state; this quantity is highly correlated with the corresponding measure computed over binding pocket RNA atoms (*R*_p_ = 0.94), and all conclusions are unchanged under either definition. The 3.0 Å success threshold was retained. Results are shown in Supplementary Fig. S11.

### System parameterization and setup

For each complex, the Maestro module of the Schrodinger 2025-1 suite [37] was used to assign protonation states to the RNA and ligands under physiological conditions (pH 7.4). The structures were then converted using the pdb4amber module of AMBERTools23. Considering the poor performance of the ff99bsc0 [38], ROC [39], Shaw, and LJbb force fields in previous studies, this work exclusively employed the OL3 [40] and YIL [41] force fields for RNA molecules. Ligands were parameterized using the GAFF2 force field [42]. Partial atomic charges for the ligands were generated using the Acpype program [43].

### Simulation system construction and ion environment

Each parameterized RNA–ligand complex was solvated in a TIP3P water box [44], with a water layer extending 12 Å from the solute. To systematically investigate ionic effects, gradient concentration experiments were designed using three different salt types (NaCl, KCl, and MgCl_2_). For each salt type, five concentrations were prepared: 0.05, 0.15, 0.50, 1.0, and 2.0 M. A control system was also prepared, containing only the minimal number of counter ions (Na^+^ or Cl^−^) required to neutralize the net charge of the solute.

To ensure precise and reproducible ion concentrations across systems of varying volumes, the exact number of ions required to achieve a target molar concentration (*C*) was calculated based on the molar concentration of pure water (55.5 mol/l). The general formula is:


\begin{eqnarray*}
\begin{array}{*{20}{c}} {{\mathrm{Number\ of\ ions = {\mathit{ C}} \times }}{{N}_{\mathrm{water}}}{\mathrm{ \div 55}}{\mathrm{.5}} {\mathrm{(S1)}}} \end{array}
\end{eqnarray*}

where: *C* is the target molar concentration of the salt (mol/l). *N*_water_ is the total number of water molecules in the simulation box.

For electrolytes with different valencies, the number of individual positive and negative ions was determined as follows:


\begin{eqnarray*}
\begin{array}{*{20}{c}} {{{N}_{\rm N{{a}^{\mathrm{ + }}}{\mathrm{/}}{{\rm K}^{\mathrm{ + }}}}}{\mathrm{ = }}{{N}_{\rm C{{l}^{\mathrm{ - }}}}}{\mathrm{ = \mathit{ C} \times }}{{N}_{\mathrm{water}}}{\mathrm{ \div 55}}{\mathrm{.5}} {\mathrm{\ (S2)}}} \end{array}
\end{eqnarray*}

\begin{eqnarray*}
\begin{array}{*{20}{c}} {{{N}_{\rm M{{g}^{{{2 + }}}}}}{\mathrm{ = \mathit{ C} \times }}{{N}_{\mathrm{water}}}{\mathrm{ \div 55}}{\mathrm{.5;}}\quad {{N}_{\rm C{{l}^{\mathrm{ - }}}}}{\mathrm{ = 2 \times }}{{N}_{\rm M{{g}^{{\mathrm{2 + }}}}}} {\mathrm{(S3)}}} \end{array}
\end{eqnarray*}

All ions were added using the tleap module in AmberTools23.

### Molecular dynamics simulation protocol

All MD simulations were conducted using the pmemd.cuda [45, 46] engine of AMBER22. To ensure the structural integrity of the highly charged RNA systems, particularly at elevated ion concentrations, a meticulous multi-stage protocol involving gradual restraint relaxation and extended equilibration was implemented.

The process began with a four-stage energy minimization procedure, which was uniformly applied to all systems. Each stage consisted of 10 000 steps of the steepest descent method followed by 10 000 steps of the conjugate gradient algorithm, using a 10.0 Å non-bonded cut-off. The harmonic restraints were progressively relaxed through this process, starting with a strong restraint of 100.0 kcal mol^−1^ Å^−2^ on all solute atoms, followed by 50.0 kcal mol^−1^ Å^−2^ on all solute heavy atoms. In the third stage, a selective restraint of 20.0 kcal mol^−1^ Å^−2^ was applied only to the RNA backbone and ligand heavy atoms. The protocol concluded with a final minimization stage that was specifically tailored to the dataset: for systems from Datasets I and II, this step was performed unrestrained, whereas a restraint of 10.0 kcal mol^−1^ Å^−2^ was maintained for systems from Dataset III and the Mg^2+^ case study.

Following minimization, each system was gradually heated in the NVT ensemble from 0.1 K to 300 K over 100 ps, using a 1 fs time step and a Langevin thermostat (gamma_ln = 2.0), while all heavy atoms were restrained at 100.0 kcal mol^−1^ Å^−2^. Subsequently, a two-phase NPT equilibration was performed. The first 100 ps phase served to equilibrate the system density at 1.0 atm, employing a Berendsen barostat (taup = 1.0 ps) with strong restraints of 200.0 kcal mol^−1^ Å^−2^. In the second 100 ps pre-equilibration phase (taup = 2.0 ps), the restraint strength was also critically tailored: for systems in Datasets I and II, restraints were relaxed to a minimal 2.0 kcal mol^−1^ Å^−2^, whereas a moderate restraint of 10.0 kcal mol^−1^ Å^−2^ was maintained for Dataset III and the Mg^2+^ case study.

Finally, production simulations were carried out in the NPT ensemble (300 K, 1.0 atm) with a 2 fs time step. For Dataset I, a 10 ns production trajectory was generated per system to systematically examine how sampling length affects predictive performance; these trajectories were partitioned into five consecutive, non-overlapping 2 ns windows (0–2, 2–4, 4–6, 6–8, and 8–10 ns) for analysis (Fig. 3A). Guided by the finding that ranking accuracy peaks within an early, ion-dependent 2 ns window and does not improve—indeed often degrades at longer times—the final protocol adopted a single 2 ns production window for Datasets II and III, which also confers a substantial throughput advantage for large-scale screening. Trajectories were saved every 2 ps, yielding 1000 frames per 2 ns window for subsequent analysis. Critically, the production protocol was specifically adapted to the scientific question addressed by each dataset. For the binding affinity predictions of Datasets I and II, simulations were performed completely unrestrained to sample the natural conformational dynamics of the complexes. In contrast, for the pose prediction evaluation using Dataset III, a gentle restraint of 5.0 kcal mol^−1^ Å^−2^ was applied to the RNA backbone atoms. This was essential to assess the stability of the initial decoy pose rather than its diffusion to a different state. Lastly, for the Mg^2+^-binding site analysis, a stronger restraint of 50.0 kcal mol^−1^ Å^−2^ was imposed on the RNA backbone to provide a stable reference frame, thereby facilitating a clear characterization of Mg^2+^ ion occupancy.

### Binding free energy calculation

#### MM/GBSA and MM/PBSA formalism

The binding affinity was estimated using the MM/GBSA and MM/PBSA methods via the MMPBSA.py script in AmberTools23. The binding free energy (Δ*G*_bind_) is calculated as the difference between the free energy of the complex and the free energies of the individual receptor and ligand:


\begin{eqnarray*}
\begin{array}{*{20}{c}} {{\mathrm{\Delta }}{{G}_{\textit{bind}}}{\mathrm{ = }}{{G}_{\textit{complex}}}{\mathrm{ - }}{{G}_{\textit{receptor}}}{\mathrm{ - }}{{G}_{\textit{ligand}}} {\mathrm{(S4)}}} \end{array}
\end{eqnarray*}

The free energy (*G*) for each species (complex, receptor, and ligand) is calculated as:


\begin{eqnarray*}
\begin{array}{*{20}{c}} {{\mathrm{G = }}{{E}_{MM}}{\mathrm{ + }}{{G}_{\textit{solv}}}{\mathrm{ - TS}} {\mathrm{(S5)}}} \end{array}
\end{eqnarray*}

where: *E*_MM_ is the molecular mechanics energy from the force field, including internal (bond, angle, dihedral) and non-bonded (van der Waals, electrostatic) interaction energies. *G*_solv_ is the solvation free energy, separated into polar and non-polar contributions. TS is the conformational entropy contribution to the free energy at temperature *T*.

#### Solvation models and dielectric constants

This study employed the GB model (igb = 8), which performed best in our previous work, and the PB model. A gradient of internal dielectric constants (ε_in_) was tested: 1, 2, 4, 8, 12, 16, and 20. To accurately model the solution properties, the external dielectric constant (ε) was not fixed at 80 but was instead calculated as a function of salt concentration using the empirical formula from Lei *et al*. [47]:


\begin{eqnarray*}
\begin{array}{*{20}{c}} {\epsilon {\mathrm{ = }}{{\epsilon }_{\mathrm{0}}}{\mathrm{ - }}{{a}_{\mathrm{1}}}{\mathrm{C + }}{{a}_{\mathrm{2}}}{{C}^{{\mathrm{3/2}}}} {\mathrm{(S6)}}} \end{array}
\end{eqnarray*}

where: ε₀ is the baseline dielectric constant of pure water (78.65), *C* is the salt concentration (mol/l), and *a*_1_ and *a*_2_ are salt-specific empirical parameters.

The parameters for NaCl were set to *a*_1_ = 15.45 and *a*_2_ = 3.76, as taken from the literature [47]. The parameters for KCl (*a*_1 _= 22.37, *a*_2_ = 8.02) and MgCl_2_ (*a*_1 _= 27.62, *a*_2_ = 13.35) were both fitted from experimental data found in a separate study [48].

#### Conformational sampling and entropy evaluation

To analyze system dynamics and temporal stability, the 10 ns Dataset I trajectories were divided into five continuous, non-overlapping 2 ns windows (0–2, 2–4, 4–6, 6–8, and 8–10 ns). Within each window, 100 evenly spaced frames were extracted for free energy calculations. Configurational entropy (−TΔS) was estimated by quasi-harmonic (QH) and normal-mode (NMODE) analysis within a single 2 ns window rather than over a continuous longer trajectory; for each ionic condition, the entropy evaluation window was chosen as the one maximizing cross-system ranking (1–2 ns for MgCl_2_, 2–4 ns for NaCl, 4–6 ns for KCl; Fig. 3A). We note that entropy terms obtained from nanosecond-scale endpoint trajectories are approximate descriptors and are used here as relative corrections for cross-system ranking rather than as converged absolute thermodynamic quantities.

#### Ionic strength equivalency for multivalent ions

To ensure comparability between different ionic environments, particularly for entropy calculations, the concentration of MgCl_2_ was converted to an equivalent NaCl concentration based on ionic strength (*I*). The ionic strength is defined as:


\begin{eqnarray*}
\begin{array}{*{20}{c}} {{\mathrm{I = 1/2}}\mathop \sum \limits_i {\mathrm{(}}{{c}_i}z_i^{\mathrm{2}}{\mathrm{)}} {\mathrm{(S7)}}} \end{array}
\end{eqnarray*}

where: *c*_i_ is the molar concentration of ion *i*, and *z*_i_ is the charge number of ion *i*.

Applying this formula, the ionic strength for a 1:1 electrolyte such as NaCl is equal to its molarity, while for a 1:2 electrolyte such as MgCl_2_, it is three times its molarity:


\begin{eqnarray*}
\begin{array}{*{20}{c}} {{{I}_{\textit{NaCl}}}{\mathrm{ = }}{{c}_{\textit{NaCl}}}\quad {\mathrm{and}}\quad {{I}_{MgC{{l}_{\mathrm{2}}}}}{\mathrm{ = 3 \times }}{{c}_{MgC{{l}_{\mathrm{2}}}}} {\mathrm{(S8)}}} \end{array}
\end{eqnarray*}

Therefore, the equivalent NaCl concentration for an MgCl_2_ solution is given by:


\begin{eqnarray*}
\begin{array}{*{20}{c}} {{{c}_{\textit{NaCl}{\mathrm{(}}\textit{equiv}{\mathrm{)}}}}{\mathrm{ = 3 \times }}{{c}_{MgC{{l}_{\mathrm{2}}}}} {\mathrm{(S9)}}} \end{array}
\end{eqnarray*}

### Classification of RNA–ligand systems

To facilitate a systematic analysis of the results, the RNA–ligand systems were categorized based on several orthogonal criteria. The biological function of each RNA was annotated according to the Nucleic Acid Knowledgebase (NAKB) database [49] https://nakb.org/. The net charges of the RNA and the ligand under physiological conditions were determined using the tleap module of AmberTools23. Finally, for diagnostic purposes only, we assigned each system a dominant residue label. For the energy-minimized complexes, we carried out MM/GBSA residue-level energy decomposition and identified the single nucleotide making the largest contribution to the total binding energy; the base type of this nucleotide (e.g. guanine or adenine) was then used as a coarse label for grouping systems. We emphasize that this label is not intended as a description of the recognition mechanism. RNA–ligand recognition is typically cooperative and distributed over several nucleotides, and the per-residue decomposition indeed shows that multiple nucleotides contribute appreciably in most systems. The dominant residue label is used here solely as a simple, reproducible one-dimensional grouping variable that allows base-type-dependent trends in computational accuracy to be detected; it does not imply single nucleotide recognition, nor is it used to define the applicability domain of P-RISE.

### Mg^2+^ ion occupancy mapping

A dedicated case study was performed to predict the preferential binding sites of Mg^2+^ ions, using the RNA–theophylline complex (PDB: 1O15) as a model system [26, 50]. The complex was first placed in a solvated box containing 0.5 M MgCl_2_, with the Mg^2+^ and Cl^−^ ions randomly distributed throughout the solvent. Subsequently, a 6 ns production MD simulation was conducted. To eliminate interference from RNA backbone flexibility in ion localization, a strong restraint of 50.0 kcal mol^−1 ^Å^−2^ was applied to the RNA backbone to provide a stable reference frame. Following the simulation, the spatial distribution of ions was determined by generating a 3D occupancy map using the grid command within the cpptraj module of AmberTools [51]. To validate our simulation-based approach, the results were compared against predictions from the 3D Reference Interaction Site Model (3D-RISM) theory [50], calculated via the rism1d and rism3d.snglpnt programs in AmberTools, which serves as a theoretical benchmark for ion distribution. All structural visualizations and occupancy maps were rendered in PyMOL. In a prior alchemical FEP study of this system, three Mg^2+^ ions were manually placed around the RNA based on chemical intuition and structural considerations [26]. Although these sites were not directly resolved experimentally, they have been widely adopted as reference binding sites in subsequent computational studies. In the present work, these sites were used as reference locations for comparison with MD-derived Mg^2+^ occupancy maps.

### Data-driven and hybrid model construction

To investigate the value of physical priors under data-scarce conditions, we constructed a hierarchical analysis framework spanning purely data-driven to physics-augmented models, covering feature engineering, model construction, hyperparameter optimization, and interpretability analysis.

#### Feature space construction and selection

To comprehensively capture molecular recognition features, we constructed two complementary and conceptually distinct feature sets. First, using RDKit [52], BioPython [53], MOE2024 (Molecular Operating Environment) [54], and ViennaRNA [55], we analyzed the ligand, receptor, and complex, respectively. We calculated a broad range of physicochemical descriptors (e.g. LogP, TPSA, molecular weight), forming the “Pure AI” feature set. To introduce physical priors, we utilized the AmberTools23 suite to extract high-dimensional dynamic parameters, constituting the “Phys-AI” feature set. Specifically, we used the cpptraj module to analyze MD trajectories and extract structural indices reflecting complex dynamic stability [e.g. RMSD and root mean square fluctuation (RMSF)], as well as various energy components output during the P-RISE calculation process (electrostatics, van der Waals, polar solvation energy, etc.). Consistent with the principle of “garbage in, garbage out”, only energy terms computed using the RNA-optimized P-RISE protocol were retained, while standard implicit solvent parameters not calibrated for RNA were excluded. To ensure feature orthogonality, the initial 146-dimensional feature space underwent a stringent two-stage filtering procedure. Zero-variance features were first removed, followed by collinearity reduction based on the Pearson correlation matrix. For highly correlated feature pairs (|*r*| > 0.9), only the descriptor with the clearest physical interpretability was retained. This procedure yielded a final set of 21 Pure AI features and 32 Phys-AI features.

#### Machine-learning algorithm implementation and training

Given the data-poor regime characterizing this study, deep learning architectures requiring large-scale datasets were excluded. Instead, five classical ML algorithms with well-documented robustness in small-sample settings were selected for comparative analysis: XGBoost (XGB), Random Forest (RF), Gaussian Process Regression (GPR), Support Vector Machine (SVM), and Multi-Layer Perceptron (MLP). All models were implemented in a Python environment; the XGB model was built using the xgboost library [56], while the other four models were built using scikit-learn [57]. To mitigate overfitting associated with high-dimensional feature spaces under limited data, we employed a conservative grid-search-based optimization strategy. Rather than simply maximizing fit on the training set, hyperparameters were selected to balance training accuracy and generalization, as evaluated by leave-one-out cross-validation (LOOCV). The hyperparameter search trajectories and convergence robustness were visualized using parallel coordinates plots (Supplementary Fig. S12).

#### Learning curve extrapolation and feature attribution

To quantify gains in data efficiency conferred by physical information, we conducted a subsampling-based learning curve analysis. Training subsets of fixed sizes (*N* = 5, 10, 15, 20, and 24) were randomly sampled, and 50 independent repetitions were performed at each size to estimate the mean and standard deviation (SD) of model performance (*R*_p_). Performance trajectories were subsequently fitted using an inverse power-law model [58]:


\begin{eqnarray*}
\begin{array}{*{20}{c}} {{{R}_p}\left( N \right) = L - a{{N}^{ - b}} \left( {S10} \right)} \end{array}
\end{eqnarray*}

where *L* denotes the asymptotic performance limit and *b* characterizes the learning rate.

Solving this relationship allowed us to estimate the theoretical sample complexity required for data-driven models to reach the zero-shot performance baseline of the purely physics-based P-RISE approach. This extrapolation is intended to compare relative data efficiency rather than to provide precise numerical predictions. Accordingly, results should be interpreted as qualitative or semi-quantitative indicators.

For model interpretability, SHAP values were computed using the shap library [59], and Beeswarm plots were used to assess whether model predictions were primarily driven by chemically intuitive descriptors or by physically grounded energy terms.

#### Energy landscape and conformational scoring assessment

To further assess the conformational scoring behavior of hybrid models, the best-performing Phys-GPR model was applied to the same candidate poses evaluated by P-RISE. Because the 20 receptor–ligand systems span markedly different absolute free energy ranges, raw predicted energies are not directly comparable. We therefore calculated a system-wise relative energy for each method:


\begin{eqnarray*}
\begin{array}{*{20}{c}} {{{E}_{{\mathrm{norm}}}} = E - {{E}_{{\mathrm{min}}}} {\mathrm{(S11)}}} \end{array}
\end{eqnarray*}

where *E*_min_ is the lowest score among candidate poses of the same system.

Relative energies were plotted against ligand RMSD to visualize pose-dependent score separation. This referencing removes the arbitrary system-specific energy offset while preserving the magnitude of pose-dependent energetic differences within each method. For P-RISE, the score corresponds to a physics-based interaction energy expressed in kcal mol^−1^. For Phys-GPR, the score is the Gaussian process regression prediction trained on experimental binding free energies (Δ*G*), whose labels span −4.3 to −10.16 kcal mol^−1^; its output therefore also lies on an energy scale rather than an arbitrary dimensionless score.

The conformational space was discretized into 1 Å RMSD bins, and the mean relative energy profile was computed for each bin. To quantify the global score–RMSD association, we report the coefficient of determination (*R*^2^) from linear regression and the Spearman-rank correlation coefficient (ρ) between Δ*E* and RMSD across pooled poses. Because *R*^2^ is invariant to linear rescaling of the score axis and ρ is invariant to monotonic rescaling, these statistics provide a scale-robust description of the score–RMSD relationship. Pose-level performance was further assessed by the Top-1 pose selection success rate, defined as the proportion of systems for which the lowest-scoring pose had an RMSD below 3 Å from the experimental pose.

### Data statistics and analysis

The computational performance in this study was evaluated using a well-defined set of statistical metrics and further interpreted through structural analyses to elucidate the underlying physical mechanisms. For binding affinity prediction, two complementary statistical indicators were used to assess predictive performance. First, the Pearson correlation coefficient (*R*_p_) quantifies the linear relationship between the predicted relative binding free energies (ΔΔ*G*_calc_) and the experimental values (ΔΔ*G*_exp_). Second, the root mean square error (RMSE) measures the absolute deviation between predicted and experimental values, providing a direct indication of prediction accuracy. For binding pose discrimination, we employed the Top–1 success rate as the evaluation criterion. This metric measures the proportion of cases in which the model correctly identifies the native (or lowest–RMSD) conformation as the most energetically favorable among a set of decoy and native poses. The final success rate is reported as the percentage of correctly identified cases relative to the total number of test samples. The prediction is judged successful if the RMSD between the model-predicted lowest-energy conformation and the crystal structure is <3.0 Å. Throughout this work we treat predictive correlation (*R*_p_) as a necessary but not sufficient indicator of model quality: a high correlation demonstrates predictive utility but does not, by itself, establish that the underlying physical mechanism has been correctly reproduced. Accordingly, mechanistic interpretations are advanced only where they are supported by independent structural or physical evidence—such as ion-site localization, per-residue energy decomposition, and binding pocket integrity analysis—rather than by correlation alone. In addition to statistical validation, structural analyses of MD trajectories were performed to probe the underlying physical mechanisms. Specifically, the RMSD of key atomic groups was calculated to monitor conformational stability during simulations, and per–residue energy decomposition was conducted to characterize the interactions of critical residues. Furthermore, for specific RNA systems, we utilized the Barnaba software [60] to calculate the eRMSD [61]. Compared with conventional RMSD, the eRMSD measures the topological similarity of base–base interaction networks, allowing it to more sensitively capture structural changes related to RNA function. Through these analyses, we aimed to gain atomic–level insights into how factors such as ionic conditions and solvent models influence the specific interaction patterns between RNA and ligands.

## Results

### Benchmark design defines the scope of RNA–ligand prediction tasks

To evaluate RNA–ligand prediction across distinct use cases, we assembled a multi-tier benchmark comprising three functionally complementary datasets. Dataset I contains 27 curated RNA–ligand complexes spanning diverse RNA functions, ligand charge states, and binding affinities (detailed in Supplementary Fig. S3 and Supplementary Tables S1 and S2), and served exclusively as the domain characterization set: it is the set on which the physical determinants of reliable prediction were identified and on which the applicability domain was subsequently defined and frozen. No parameter or domain boundary was adjusted using any of the external data described below. Performance on this dataset was assessed using the Pearson correlation coefficient (*R*_p_), which quantifies relative affinity ranking, and the RMSE, which captures the absolute deviation between calculated and experimental binding free energies.

We next used independent tasks to test whether the resulting framework transfers beyond the primary benchmark. Because RNA conformational stability is strongly shaped by divalent ions, we included an Mg^2+^ localization case study to examine whether the conformational preparation strategy can recover functionally relevant ion-binding sites. Dataset II was assembled as an external validation set and partitioned by role according to a domain criterion that had been fixed beforehand on Dataset I and that depends only on two *a priori* computable quantities, the net charges of the ligand and of the RNA (see the Materials and methods): the within-domain subsets (Benzimidazole and Rbt) test reproducibility on chemotypes distinct from Dataset I, whereas the out-of-domain Flavins subset tests generalization beyond that domain, its ligand net charge lying outside the entire charge range spanned by Dataset I. Both were evaluated using *R*_p_ and RMSE. Dataset III was designed for pose discrimination, namely ranking near-native binding modes against computational decoys; performance on this task was evaluated by the Top-1 success rate.

Finally, to place these physics-based results in the context of current RNA data constraints, we established a comparative ML benchmark using both descriptor-only and physics-augmented feature sets. This analysis was used not to optimize P-RISE itself, but to evaluate how far current data-driven models can approach a physically grounded reference under limited data conditions.

### RNA-binding pocket integrity under ionic conditions determines predictive stability

Reliable endpoint energy evaluation requires a conformational ensemble that preserves the RNA-binding pocket. For highly flexible and charged biomacromolecules such as RNA, the generation of a stable and physically plausible conformational ensemble poses the primary challenge. Consequently, our research commences with an in-depth analysis of two pivotal factors intricately linked to structural integrity in Dataset I: the ionic environment and the duration of MD simulations.

#### Ionic environment preserves RNA-binding pocket integrity and improves predictive accuracy

Because RNA structure is highly sensitive to ionic conditions, we first examined how salt identity and concentration influence the conformational stability required for reliable energy evaluation. Abundant literature has firmly established that the local ionic concentration of RNA within cells is exceedingly high, with Mg^2+^ playing a particularly pivotal role [6, 62, 63]. We systematically investigated the effects of NaCl, KCl, and MgCl_2_ at various concentrations.

Across Dataset I, performance depended strongly on both force field choice and ionic treatment (Fig. 2; Supplementary Figs S4 and S5). Under the conditions tested here, the contemporary OL3 force field consistently and markedly outperforms the YIL force field, which shows little sensitivity to alterations in the ionic environment. Moreover, the GBSA implicit-solvent model far outshines the PBSA model (the subpar performance of the latter might be attributed to its parameters being predominantly optimized for NaCl). Specifically, in explicit-solvent simulations, the predictive accuracy of the OL3 force field escalates substantially with increasing NaCl concentration, whereas the YIL force field remains largely impervious to changes in ion concentration. Together, these results indicate that explicit representation of the ionic microenvironment is a prerequisite for reliable RNA–ligand energy evaluation.

**Figure 2.**
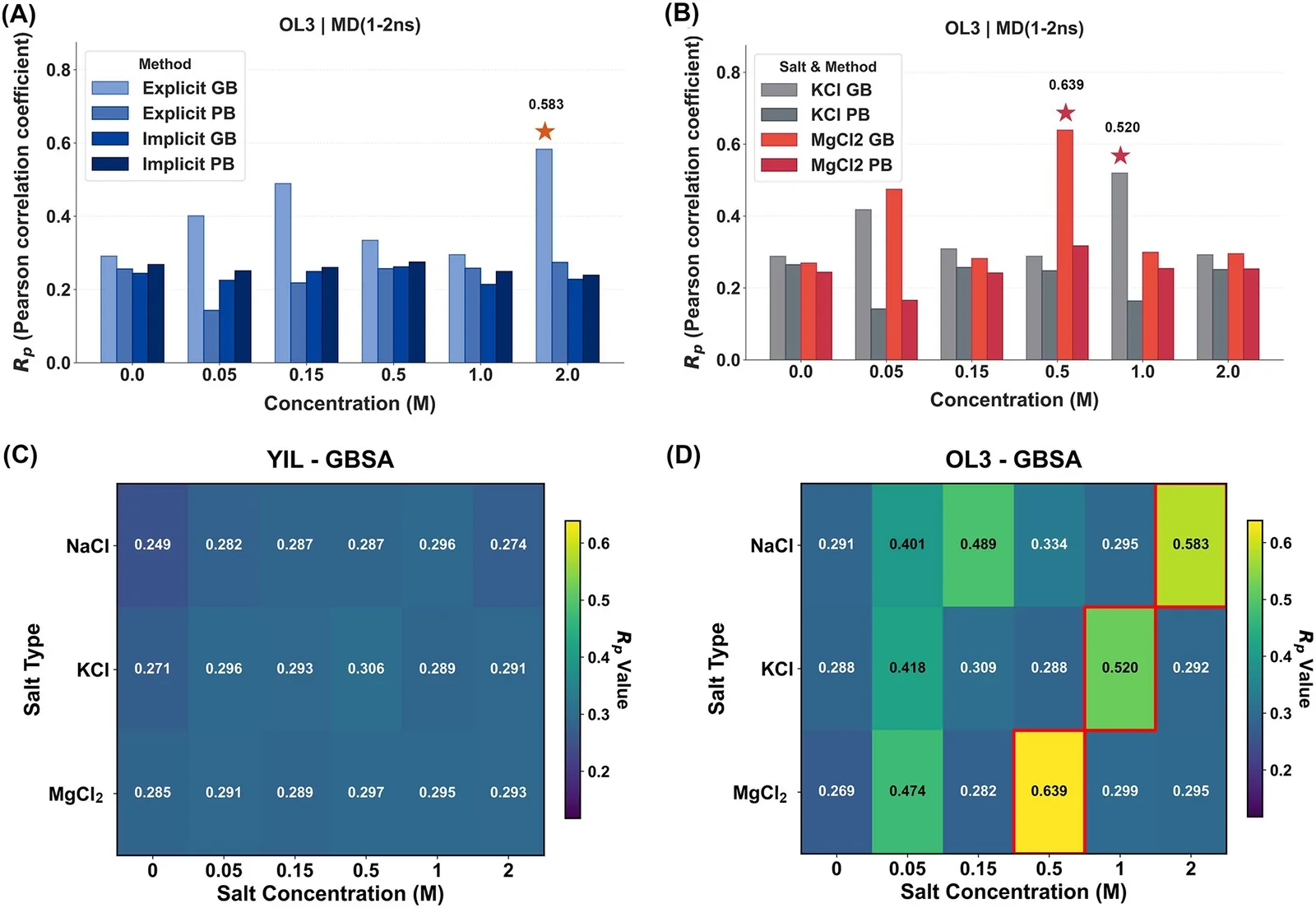
Traversal of ionic environment. (**A**) Pearson correlation (*R*_p_) values for explicit and implicit simulation in OL3 force fields under varying NaCl concentrations using GBSA and PBSA models. (**B**) *R*_p_ values for the OL3 force field under different ion types (KCl and MgCl_2_) and concentrations. The red stars on the bar chart represent the best performance under the current ion. (**C** and** D**) Heatmaps showing *R*_p_ as a function of concentration for the YIL and OL3 force field with GBSA models, respectively. The GB model under the OL3 force field has the best effect.

Within this framework, the predictive accuracy exhibits a remarkable sensitivity to specific ionic conditions. As depicted in the bar chart of Fig. 2A, for NaCl, the accuracy improves with increasing concentration, reaching a peak at 2 M. In the assessment of KCl and MgCl_2_ (Fig. 2B), we observe that altering the ion type indeed exerts a positive influence on predictive accuracy. As anticipated, 0.5 M MgCl_2_ yields the highest predictive correlation (*R*_p_ = 0.639), consistent with the well-established stabilizing role of Mg^2+^ in RNA folding. We emphasize that this optimal condition should be understood not as a reproduction of physiological bulk ionic strength, which these concentrations exceed, but as an effective electrostatic scaffold that stabilizes the RNA fold and, critically, the local binding pocket. This interpretation is moreover physically reasonable: because RNA is a densely charged polyanion, counterion condensation renders the local ion concentration in the immediate vicinity of the backbone far higher than the bulk value—by up to roughly three orders of magnitude for Mg^2+^ around RNA [63]. Elevated explicit salt conditions may therefore provide a more faithful description of the ionic microenvironment actually experienced by the binding pocket, rather than a less realistic one. In comparison, 1 M KCl also demonstrates a notable enhancement, with an *R*_p_ value of 0.520, which surpasses the optimal value achieved in a pure water environment (*R*_p_ = 0.513) [24]. To gain a more comprehensive insight into this trend, we plotted heat maps of *R*_p_ values as a function of concentration (Fig. 2C, D; Supplementary Fig. S5). These visualizations clearly indicate that under the OL3/GBSA model, all ions exhibit a minor performance apex near the physiological concentration of ~0.15 M. However, further increasing the concentration can further refine the results. Significantly, all three optimal ionic conditions—0.5 M MgCl_2_, 2 M NaCl, and 1 M KCl—exhibit predictive performances that far exceed the best outcomes attainable in a pure water environment [24]. Together, these findings indicate that explicit representation of the ionic microenvironment is important for improving predictive accuracy in RNA–ligand free energy evaluation.

#### Simulation duration defines a finite window of physically informative sampling

Having identified the optimal ionic environment capable of serving as a “structural scaffold”, our subsequent investigation delved into the influence of simulation duration. This was aimed at uncovering how the stabilizing effect of the ionic environment translates into enhanced accuracy. For each system, we generated 10 ns trajectories and computed the binding energy across five consecutive 2 ns windows: 0–2, 2–4, 4–6, 6–8, and 8–10 ns. The analysis results are presented in Fig. 3A. These results unveil a non-monotonic relationship that is contingent upon the type of ion: rather than increasing monotonically with simulation time, the prediction accuracy reaches a peak within specific time windows. Specifically, for MgCl_2_, the peak occurs at 1–2 ns; for NaCl, at 2–4 ns; and for KCl, at 4–6 ns. Thereafter, the accuracy begins to decline, indicating that simulations of excessive duration can actually cause deterioration of the results.

**Figure 3.**
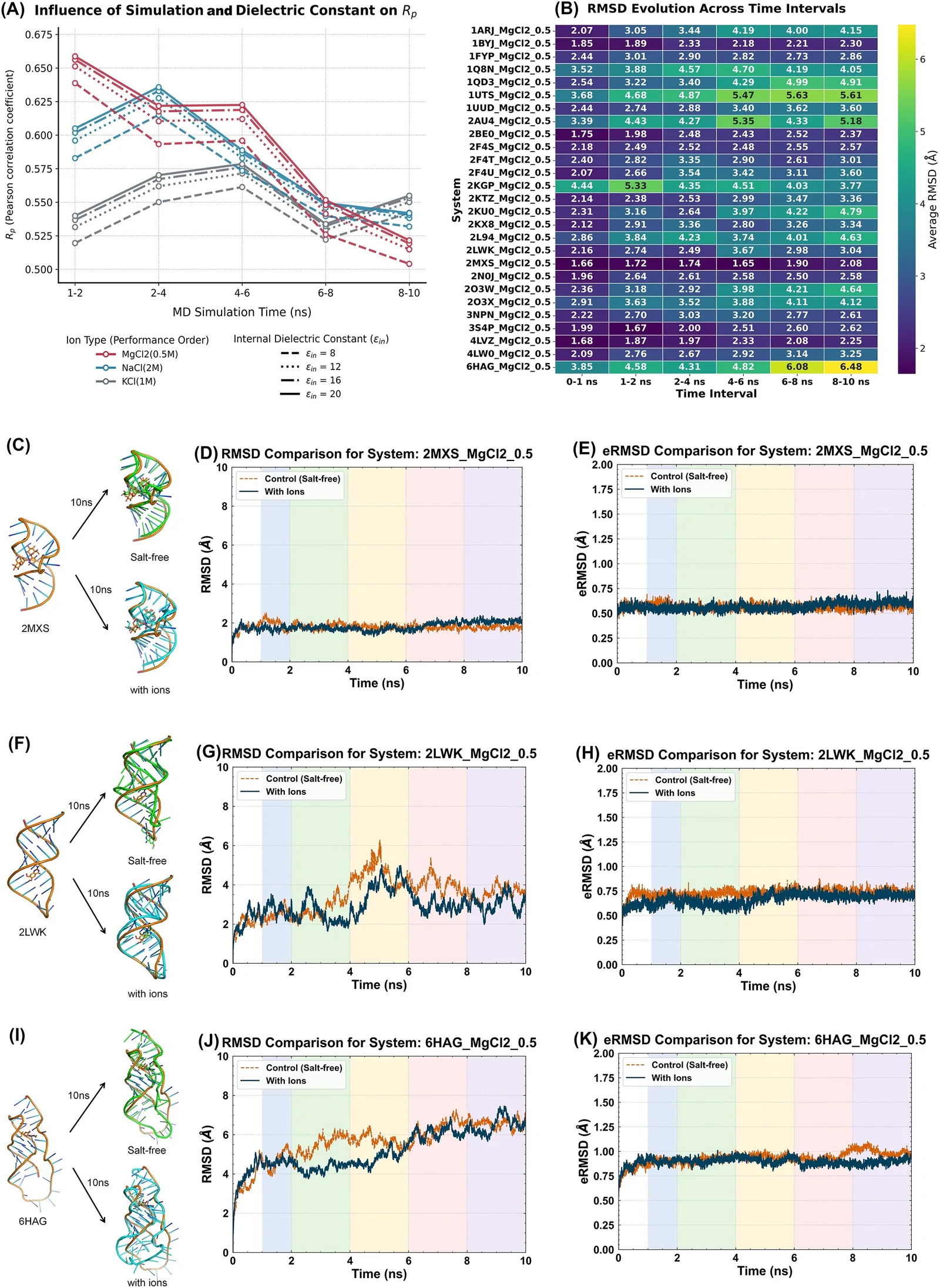
The dual effect of simulation duration on predictive accuracy and the role of ions in maintaining RNA structural stability. (**A**) Prediction accuracy, measured by the *R*_p_, as a function of the MD simulation time window under optimal ion conditions for MgCl_2_, NaCl, and KCl. The analysis reveals a non-monotonic relationship, where peak accuracy is achieved within a finite “optimal time window” that varies by ion type. (**B**) Average RMSD of all 27 systems in 0.5 M MgCl_2_. (C–K) Detailed structural analyses of three representative systems illustrating distinct stability patterns. For all structural overlays, crystal structures are shown in orange, pure water simulations in green, and 0.5 M MgCl_2_ simulations in blue. (C, D, E) Analysis of an inherently stable system (2MXS). The structural overlay (**C**), RMSD plot (**D**), and eRMSD plot (**E**) all show high stability across all conditions, indicating a well-behaved system where the force field performs adequately. (F, G, H) Analysis of an ion-dependent system (2LWK). The structural overlay (**F**) shows catastrophic unfolding and ligand dissociation in pure water, whereas the structure is preserved in 0.5 M MgCl_2_. This observation is quantified by the dramatic increase in RMSD (**G**) and eRMSD (**H**) in the pure water simulation. (I, J, K) Analysis of the 6HAG system, highlighting the critical role of local stability. Despite significant global flexibility shown in the structural overlay (**I**) and quantified by large fluctuations in RMSD (**J**) and eRMSD (**K**), the ligand remains stably bound. This demonstrates that the stability of the local binding pocket, rather than global rigidity, is the key determinant for stable ligand binding.

To understand the molecular basis of this “optimal time window” phenomenon, we analyzed the structural stability of the RNA backbone. Using the globally optimal condition of 0.5 M MgCl_2_ as an exemplar, we calculated the time RMSD of RNA backbone atoms for all 27 systems within the benchmark set and compared these with the pure water environment. As illustrated in Fig. 3B and Supplementary Fig. S6, the average RMSD across all systems is substantially lower and more stable in 0.5 M MgCl_2_, confirming the superior stabilizing effect of the ionic environment. However, a deeper analysis reveals that the systems exhibit three distinct behavioral patterns:

**Inherently stable systems (e.g. 2MXS)**. In a subset of complexes, the initial RNA fold is intrinsically stable. The complex’s structure remains well maintained throughout the 10 ns simulation, showing little deviation from the crystal structure in both pure water and 0.5 M MgCl_2_ (Fig. 3C). Correspondingly, both the RMSD and eRMSD (a metric for RNA structural similarity based on base–base interaction networks) show high stability (Fig. 3D, E). This indicates that for such systems, the force field itself can adequately describe the native conformation.

**Ion-dependent stabilization (e.g. 2LWK)**. In these systems, the stabilizing effect of ions is paramount. In the pure water environment, the RNA structure in 2LWK undergoes significant conformational deviation, leading to the complete dissociation of the ligand from its binding pocket (Fig. 3F). This structural collapse is quantified by a sharp increase in both RMSD and eRMSD (Fig. 3G, H). In stark contrast, the core tertiary fold and ligand-binding mode are effectively preserved throughout the simulation in 0.5 M MgCl_2_. This directly demonstrates that Mg^2+^ provides the necessary structural support for stable ligand binding by stabilizing the RNA’s native fold. Similar stabilizing effects were consistently observed under the optimal NaCl and KCl conditions (Supplementary Figs S7 and  S8).

**The pivotal role of local stability (e.g. 6HAG)**. Most interestingly, we observed that global rigidity is not a prerequisite for stable ligand binding. In systems such as 6HAG, the ligand remains stably bound within its pocket despite significant global RMSD and eRMSD fluctuations (Fig. 3I–K). Further analysis demonstrated that the RNA’s flexibility primarily originated from regions distal to the binding pocket, which were inherently disordered in the crystal structure. In contrast, the local structure of the binding pocket maintained remarkable stability in the ionic environment. This critical finding refines our conclusion: for the prediction of binding free energy, it is the structural stability of the local binding pocket, not the global rigidity of the entire macromolecule, that is the paramount factor.

Together, these results lead to our first key finding: the principal contribution of high ionic strength is to act as an electrostatic scaffold that provides a high-quality conformational ensemble for free energy calculations by inhibiting structural instability, particularly within the binding pocket region. We stress that this stabilization is selective rather than global: as the 6HAG system illustrates (Fig. 3I–K), the macromolecule retains substantial global conformational flexibility even under 0.5 M MgCl_2_, and only the local pocket is preferentially stabilized. The improved scoring consistency therefore reflects restraint of force-field-driven local drift, not an artificial freezing of the RNA as a whole. However, the decline in accuracy after long simulations suggests that even the best available force fields exhibit subtle imperfections, rooted in part in their fixed-charge, non-polarizable approximation of a strongly electrostatic RNA ion environment; such force field artifacts accumulate over time, driving the conformational ensemble toward non-physical states. Consequently, this ion-type-dependent, finite “optimal simulation length” represents a delicate balance between “sufficient sampling to capture conformational dynamics” and “avoiding the accumulation of force field errors”.

### Coupled electrostatic and entropic corrections improve reliable RNA–ligand energy evaluation

Having established a strategy for generating a stable conformational ensemble, our attention turned to refining the energy model itself. This entailed a systematic investigation of two central components within the MM/GBSA free energy equation in Dataset I: the enthalpy contribution, predominantly governed by the dielectric constant, and the solute entropy contribution −TΔS, which is frequently overlooked.

#### Electrostatic treatment defines an effective error cancellation regime

ε_in_ and ε_out_ are essential parameters for mimicking the electrostatic shielding effects of the solute and solvent, respectively. Theoretically, a high ion concentration can alter the macroscopic dielectric properties of the solvent via the screening effect. Thus, for the three optimal ionic conditions identified in the previous section, we systematically explored the combined effects of ε_in_ (scanned from 1 to 20) and ε_out_ (80 versus fix).

To decouple the influence of the parameters themselves from the noise associated with structural sampling, we initially compared the disparities between using solely the single-frame conformation following energy minimization and using the ensemble conformations from short-term MD calculations. The results in Fig. 4 show a clear difference between static and ensemble-based evaluations. When employing the single-frame conformation, the *R*_p_ values obtained from all parameter combinations were subpar and exhibited no substantial differences (Fig. 4A), underscoring the limitations of static structures in depicting dynamic binding processes. Conversely, when we utilized the ensemble conformations from 1–2 ns short-term simulation trajectories, the performance distinctions among different parameter settings became immediately evident (Fig. 4B). As per the findings above, the 1–2 ns period represents the time of maximum structural stability for all systems.

**Figure 4.**
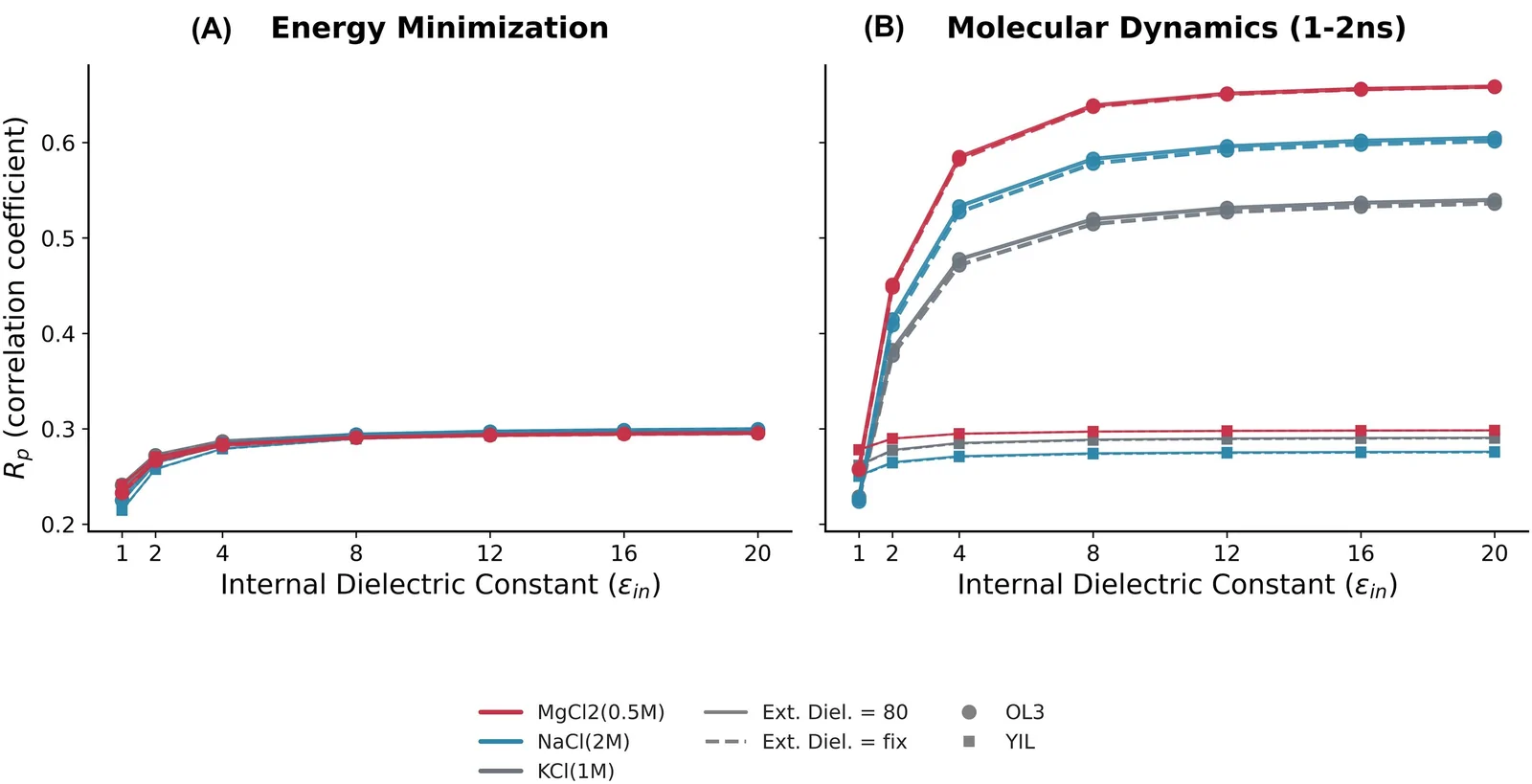
Effect of dielectric constants (ε_in_ and ε_out_). (**A**) The performance of *R*_p_ when energy is minimized. (**B**) The performance of *R*_p_ in 1–2 ns trajectories. ε_in_ and MD trajectories have a significant impact on the enhancement of the *R*_p_ value.

A clear outcome of this analysis is that predictive performance is highly sensitive to the choice of the ε_in_. For the high-performing OL3 force field, increasing the value of ε_in_ from 1 to 20 leads to a significant and continuous improvement in the predictive correlation (*R*_p_). In contrast, at this stage, the choice of ε_out_ has a relatively minor impact. When ε_out_ is fixed at 80 and the function value depends on concentration, the obtained outcomes are largely comparable. Only under certain conditions does ε_out _= 80 show a slight advantage.

Based on these observations and taking into account their robustness and simplicity, we designate ε_in _= 20 and ε_out _= 80 as our “Baseline Protocol”. It is imperative to emphasize that this represents a provisional conclusion derived from an incomplete model that does not yet incorporate entropy contributions. In subsequent sections, we will further elucidate that in a more physically comprehensive model, the optimal parameters may undergo subtle alterations.

#### Entropy correction is empirically coupled with the solvent model

Next, we evaluated the impact of conformational entropy change (−TΔS), an important but computationally challenging component of the binding free energy. We emphasize at the outset that, on nanosecond-scale trajectories of highly flexible RNA, QH and NMODE entropies cannot capture slow collective motions and are therefore treated as approximate relative descriptors rather than converged thermodynamic quantities.

As shown in Fig. 5A, after applying QH correction within a single 2 ns production window (4–6 ns) drawn from a 10 ns trajectory, the predicted *R*_p_ values were significantly and systematically improved, indicating that QH entropy correction substantially improves the predictive ranking in these RNA systems. We interpret this as improved statistical consistency of the ranking rather than as a demonstration that the absolute entropy contribution of each system is individually recovered. In contrast, NMODE correction slightly reduced the correlation, which is consistent with the inherent limitations of its principle. This is because the core assumption of the NMODE method is that molecules vibrate well near a single potential energy, and it is severely inadequate in capturing conformational entropy for RNA–ligand binding processes involving significant conformational rearrangements and flexibility change. Consequently, its ability to capture conformational entropy is severely limited, introducing notable prediction errors.

**Figure 5.**
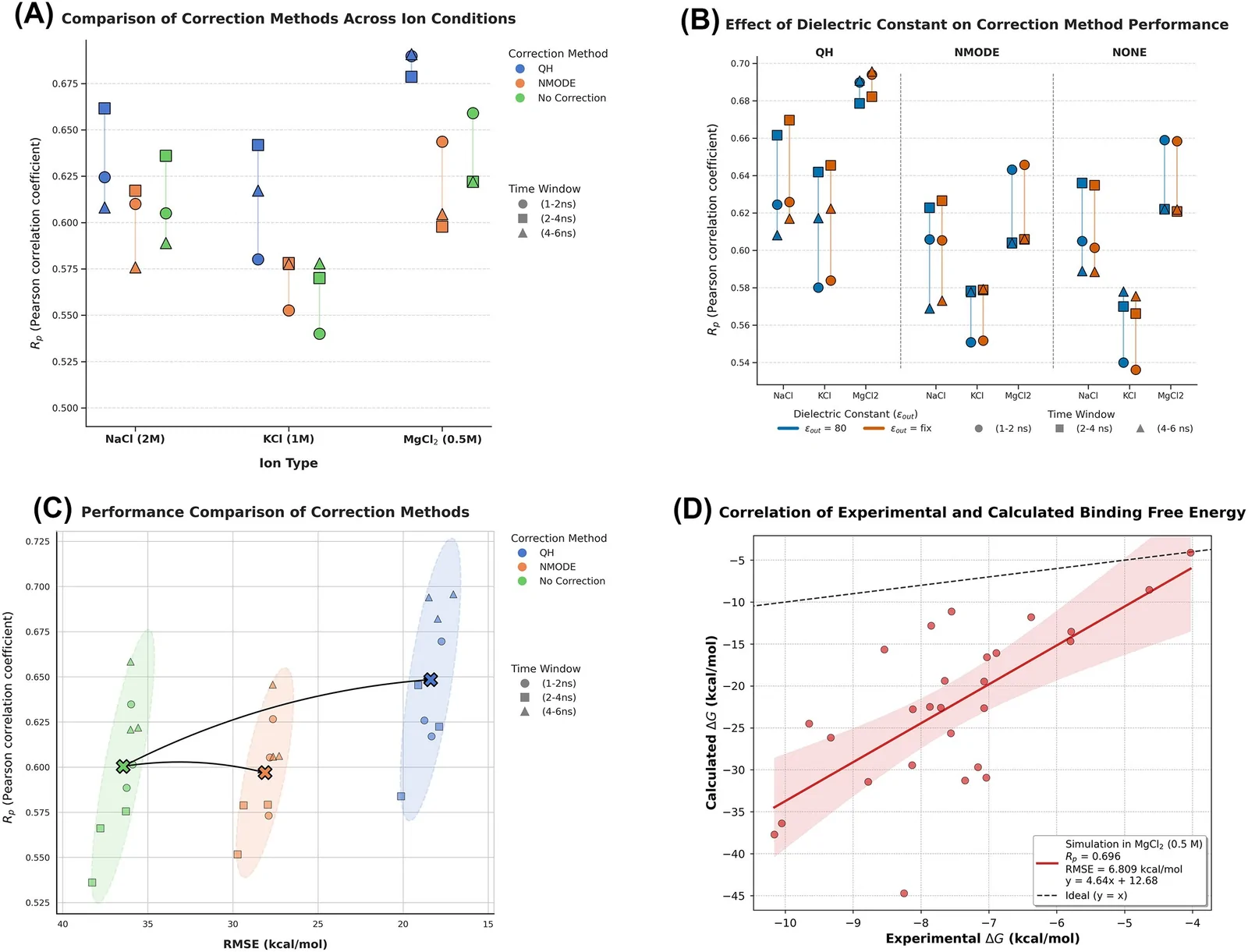
Impact of entropy correction and final protocol performance. (**A**) *R*_p_ values plotted against simulation length for protocols without entropy (green), with QH correction (blue), and with NMODE correction (orange). The QH correction effect is the best. (**B**) Performance comparison for ε_out _= 80 versus fix. Under the condition ε_out _= fix, the application of QH and NMODE correction methods resulted in a moderate improvement in the *R*_p_ value. (**C**) Dual performance comparison of *R*_p_ and RMSE under three protocols. The QH correction effect is the best. (**D**) Correlation plot of calculated versus experimental binding free energies for the final P-RISE protocol, achieving *R*_p_ = 0.696.

Crucially, the introduction of entropy prompts us to re-examine the choice of dielectric constants. While ε_in _= 20 remains the best-performing choice in our benchmark, indicating that this parameter is highly robust as a correction for the internal polarization effect of the solute, an interesting reversal occurs in the influence of the external dielectric constant (Fig. 5B). After the QH entropy correction is introduced, the physically more realistic, concentration-dependent ε_out _= fix value begins to consistently outperform the fixed ε_out _= 80. This subtle shift is consistent with an error compensation mechanism, suggesting that the slight advantage of ε_out _= 80 in the entropy-free model might result from an accidental error cancellation with the neglected entropy term. Combining a more realistic entropy term (QH correction) with a concentration-dependent ε_out_ yields the most internally consistent and best-ranking protocol in our benchmark. We interpret this synergy as improved statistical robustness of the relative ranking, rather than as evidence that the absolute energetics of each term are individually recovered.

To confirm that this entropy contribution acts as a stable ranking correction rather than a noisy artifact of any single trajectory segment, we examined its variability across the five consecutive 2 ns windows that span the full 10 ns trajectory for all 27 systems. The window-to-window fluctuation of the QH −TΔS term is small and shows no systematic drift: its cross-system SD stays between 0.64 and 0.75 kcal mol^−1^ across all five windows (Supplementary Fig. S9A, B), whereas the −TΔS term itself varies over 7.9–27.0 kcal mol^−1^ between systems (between-system SD ~4.7 kcal mol^−1^). The ranking signal is therefore roughly six times larger than the segment-to-segment noise, so the correction is bounded and essentially insensitive to which window is analyzed. Consistent with this, the two windows used in this study give statistically indistinguishable performance, with *R*_p_ = 0.694 for the 1–2 ns window and *R*_p_ = 0.696 for the 4–6 ns window (Δ = 0.002). Importantly, although the per-window *R*_p_ declines beyond 6 ns (Supplementary Fig. S9C), this decline is not accompanied by any increase in the dispersion of −TΔS (Supplementary Fig. S9A, B); it instead mirrors the progressive drift of the complexes away from their crystallographic conformations at longer simulation times (Fig. 3C–K), and is thus transmitted through the enthalpic term rather than reflecting any instability of the entropy correction. We further verified that this correction is reproducible across independent trajectories rather than dependent on a single random seed. Repeating the simulations with three independent seeds, the per-system −TΔS values remain tightly clustered around their means (Supplementary Fig. S9D), with a mean per-system SD across replicates of 0.82 kcal mol^−1^, ~17% of the between-system spread. Even for the most demanding case, i.e. the 90-residue ligand-bound complex 4LW0, the −TΔS estimates are consistent across both replicates and windows (Supplementary Fig. S9E), confirming that the ranking correction is robust for the largest and most flexible systems in the benchmark.

Furthermore, the introduction of entropy also reveals the decoupling of the ranking ability (*R*_p_) and the absolute accuracy (RMSE) (Fig. 5C). A particularly important observation is that these two metrics do not change in tandem. QH correction can simultaneously improve both, whereas NMODE does not improve *R*_p_ but consistently reduces RMSE. This indicates that although NMODE cannot capture the fine differences in relative entropy changes, it can serve as an effective “average penalty term” to correct the systematic overestimation of binding energy from ignoring entropy from a macroscopic perspective.

This suite of intertwined observations—namely, the decoupling between ranking ability (*R*_p_) and absolute accuracy (RMSE), and the shift in the optimal dielectric model upon introducing entropy—collectively leads to our second key finding: the strong predictive power of endpoint methods for RNA systems does not arise from perfectly accurate modeling of each individual energy term, but instead from a subtle error cancellation mechanism. Within this mechanism, an effectively elevated high internal dielectric constant (ε_in _= 20) systematically serves as a “corrective factor”, compensating for the classical force field’s general overestimation of intramolecular electrostatic interactions in the solute. Furthermore, we observe an empirical coupling among these terms: the highest cross-system ranking is obtained only when the entropy correction (QH) is combined with a concentration-dependent ε_out_. Rather than demonstrating a fully self-consistent physical model, this regularity indicates that accounting for a solute entropy penalty complements the concentration-dependent dielectric treatment, and that the two corrections must be applied together to achieve optimal predictive accuracy. We stress that this interpretation is not a claim of mechanistically exact energy recovery. The elevated internal dielectric constant functions as an effective error cancellation device, and its justification here is empirical and statistical—improved and stabilized cross-system ranking correlation—rather than a statement about the true dielectric response of RNA. The value of endpoint scoring for RNA, in our view, lies in this reproducible cancellation of systematic errors, not in the faithful reconstruction of individual physical energy terms.

#### Integrating these determinants defines the P-RISE framework

Integrating the physical determinants identified above, we defined the P-RISE framework as follows: using the OL3 force field, simulating in a 0.5 M MgCl_2_ environment, evaluating energetics on a single 2 ns production window extracted from a 10 ns trajectory, performing energy calculations with ε_in_ = 20 and ε_out_ = fix, and finally conducting QH entropy correction. On the core benchmark Dataset I, this protocol achieves the best predictive performance with *R*_p _= 0.696, representing a substantial improvement over the previously best-achieved *R*_p_ of 0.513 by Dejun Jiang *et al*. [24]. Importantly, the earlier 1–2 ns window performs equivalently (*R*_p_ = 0.694; Δ = 0.002, statistically indistinguishable; see Supplementary Fig. S9C), while requiring only one-fifth of the sampling of a full 10 ns trajectory. We therefore adopt the 4–6 ns window as the reference protocol for reporting best-case accuracy on Dataset I, but recommend the 1–2 ns window for practical high-throughput screening, where its near-identical ranking power at a fraction of the computational cost is decisive. Datasets II and III were accordingly evaluated on the 1–2 ns window. The comparison between final computed and experimental values is shown in Fig. 5D.

To assess the reproducibility of this result, we repeated the entire Dataset I evaluation with three independent random seeds. The ranking performance is stable across replicates: the three seeds individually give *R*_p_ = 0.62 ± 0.07 (mean ± SD), and averaging the per-complex predictions over the three seeds yields *R*_p_ = 0.631, with the single-run reference value of 0.696 lying within 1 SD of the replicate mean (Supplementary Fig. S9F). Individual single-run correlation coefficients should therefore be read as carrying an uncertainty of order ± 0.07, and the reported ranking accuracy reflects a reproducible property of the protocol rather than the outcome of a single stochastic trajectory. In addition, to ensure that these conclusions are not driven by the specific composition of the benchmark, we quantified the statistical robustness of each design choice. Using paired Δ*R*_p_ comparisons across the 27 systems—so that shared noise cancels—every determinant retained in P-RISE produces a positive Δ*R*_p_ (Supplementary Table S6); the two dominant contributions, the internal dielectric treatment and the production window choice, are individually resolved at the present sample size [bootstrap 95% confidence intervals (CIs) excluding zero], whereas the ionic strength, entropy, and external dielectric terms remain directionally positive but not individually resolved at *N* = 27, consistent with their role as bounded relative-ranking corrections. A leave-one-out analysis further confirms that no single system dominates the overall correlation (seed-averaged full-set *R*_p_ = 0.631; range 0.577–0.670; Supplementary Fig. S9G).

### P-RISE transfers across RNA-relevant prediction tasks

Having established the physical determinants underlying reliable RNA–ligand prediction, we next asked whether these principles transfer across distinct RNA-relevant tasks. To address this question, we evaluated P-RISE in three independent settings: Mg^2+^ site localization, binding affinity prediction for external chemotypes, and binding pose discrimination. Together, these analyses assess the practical scope of the framework and define its current applicability boundaries.

#### P-RISE recovers functionally relevant Mg^2+^-binding sites in an RNA system

Accurate prediction of Mg^2+^ ion-binding sites is crucial for RNA research as these divalent cations directly affect the stability of RNA folding, which in turn determines the accuracy of subsequent free energy calculations. The success of this task serves not only as a key conceptual validation of methodological effectiveness but also as a direct application and verification of our first core finding—that ions act as an “electrostatic scaffold” stabilizing RNA structures. Previous studies have confirmed that accurately determining the positions of Mg^2+^ ions can significantly improve the precision of free energy calculations [27]. In this study, researchers manually placed three key Mg^2+^ ions in the RNA–theophylline complex (PDB ID: 1O15) based on existing literature, serving as a high-quality starting point for the calculation. We used these three expert-determined sites (Fig. 6A) as the benchmark for this study, aiming to test whether the automated workflow could recover these reference regions and provide a practical starting point for ion-aware RNA system preparation.

**Figure 6.**
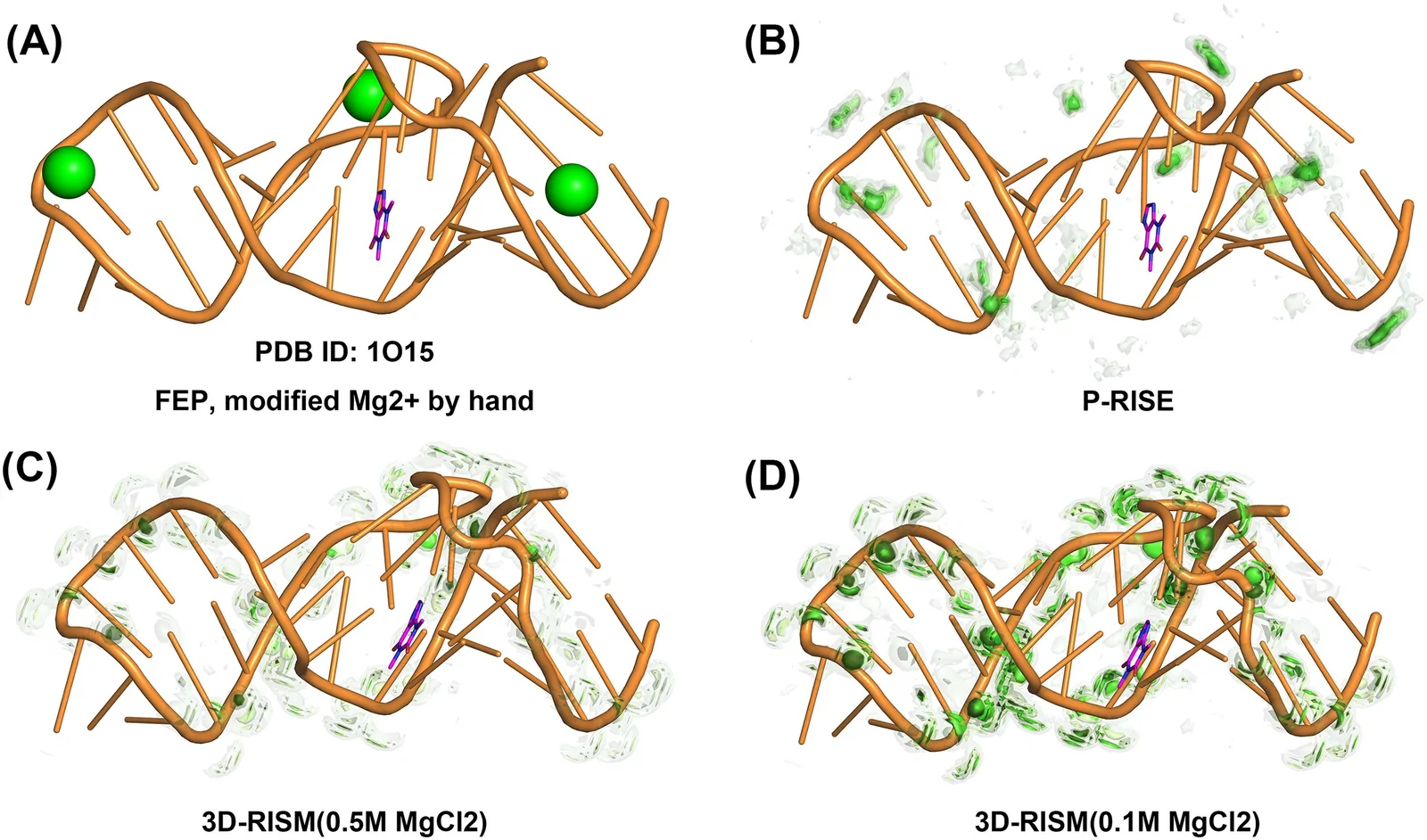
Assessment of the magnesium ion position prediction ability of P-RISE. (A–D) Prediction of Mg^2+^-binding sites in the RNA–theophylline complex (PDB: 1O15). (**A**) The experimental structure showing three magnesium ions (green spheres) placed by experts for stabilizing RNA. (**B**) Binding sites predicted by our method. (**C** and** D**) Ion density maps calculated by the 3D-RISM method using 0.5 and 0.1 M MgCl_2_, respectively. In B–D, the darker the green color, the greater the possibility that the ion is located at the current position.

As shown in Fig. 6B, the darker the green around the RNA, the more likely that location is to bind Mg^2+^ ions. It can be seen that our method not only perfectly matched all three Mg^2+^ ion sites manually placed by experts but also predicted four new binding regions with high probability. These results indicate that the method can recover the reference Mg^2+^ regions while also highlighting additional candidate sites for further inspection. In contrast, we used the “gold standard” method 3D-RISM [64, 65], which is included in the Amber official tutorial, for the same analysis. The results are shown in Fig. 6C. Under the same 0.5 M MgCl_2_ condition, the ion density distribution calculated by 3D-RISM is very diffuse and cannot clearly distinguish the exact binding sites. Even considering that high ion concentration may cause signal interference, we further reduced the MgCl_2_ concentration to 0.1 M for testing, but the results did not show significant improvement (Fig. 6D). Furthermore, we attempted to utilize another renowned predictive tool, the MCTBI webserver [66]. However, the submitted computational tasks failed to return results within a reasonable time frame, consequently precluding its inclusion in the direct comparative analysis.

By comparison, P-RISE produced a more spatially focused occupancy pattern under the tested conditions, consistent with the manually defined reference regions. This result is consistent with what we observed in Fig. 3, once again confirming the key role of Mg^2+^ in maintaining the stability of RNA structure. It also indicates that this method can serve as an efficient and automated pre-processing tool, providing a reliable and more comprehensive initial ion layout for subsequent more complex calculations (such as FEP), thereby potentially avoiding the subjectivity and omissions in manual site selection. According to a published article on FEP+ for RNA–ligand research [27], this process is necessary for the stability of FEP simulations, thus highlighting the significance of our method.

#### P-RISE defines an applicability domain for affinity prediction and generalizes to new chemotypes

Accurate prediction of ligand–receptor binding free energy is a central element of the drug discovery pipeline. In this section, we follow a strictly sequential, one-directional workflow. We first dissect the applicability boundaries of P-RISE using Dataset I alone, which leads to our third core finding and defines an applicability domain that is fixed before any external data are examined. Only then do we turn to the independent external Dataset II, which we deliberately partition into two logically distinct components: a within-domain reproducibility test (the Benzimidazole and Rbt subsets, which are structurally distinct from Dataset I yet physicochemically consistent with the defined domain) and an out-of-domain generalization test (the Flavins subset, which by design violates the domain). Because no method parameter or domain boundary is ever adjusted using Dataset II, this design precludes circularity: it separates hypothesis generation (Dataset I) from validation (Dataset II).

**Boundaries of predictive power: a categorical analysis of representative systems**. After establishing the P-RISE protocol with the highest predictive power, a more profound question emerges regarding its applicability domain. To answer this, we analyzed the benchmark Dataset I in two complementary ways. We first used the dominant residue label as a diagnostic grouping variable to test whether predictive accuracy depends on base type, an analysis aimed at exposing limitations of the underlying fixed-charge force field rather than at defining a domain of applicability. We then characterized the applicability domain proper, which is defined along two physicochemical axes only, the net charge of the ligand and the net charge of the RNA, as these are the quantities that can be evaluated *a priori* for any new system and that were subsequently used to assess domain membership.

First, we classified the systems in Dataset I based on the dominant nucleotide residue identified through energy decomposition and compared the computed values against experimental data for each category, calculating their respective *R*_p_ values. Among the four RNA bases, we observed that systems involving guanine (G) as a key interaction partner pose the greatest computational challenge, exhibiting systematically lower predictive accuracy (Fig. 7A). This reduced performance may reflect guanine’s greater structural and electronic complexity, which surpasses that of other bases. Guanine possesses the most abundant hydrogen-bonding sites, including two donors (N1-H and the C2-amino group) and three acceptors (N3, N7, and the O6 carbonyl oxygen), enabling it to form more complex and diverse interaction networks with ligands (Supplementary Fig. S10). We stress that this grouping is diagnostic rather than mechanistic: because recognition is generally cooperative and distributed across multiple nucleotides, the label identifies the largest single contributor rather than the full interaction network, and the guanine-associated accuracy loss should be read as a signal of where fixed-charge force fields struggle, most plausibly with the polarization and directional hydrogen bonding of the most electron-rich base, rather than as evidence for a distinct “single-residue recognition mode”. Consistent with this interpretation, the dominant residue label is not used as a criterion for domain membership; the applicability domain is defined solely by the ligand and RNA net charges described below.

**Figure 7.**
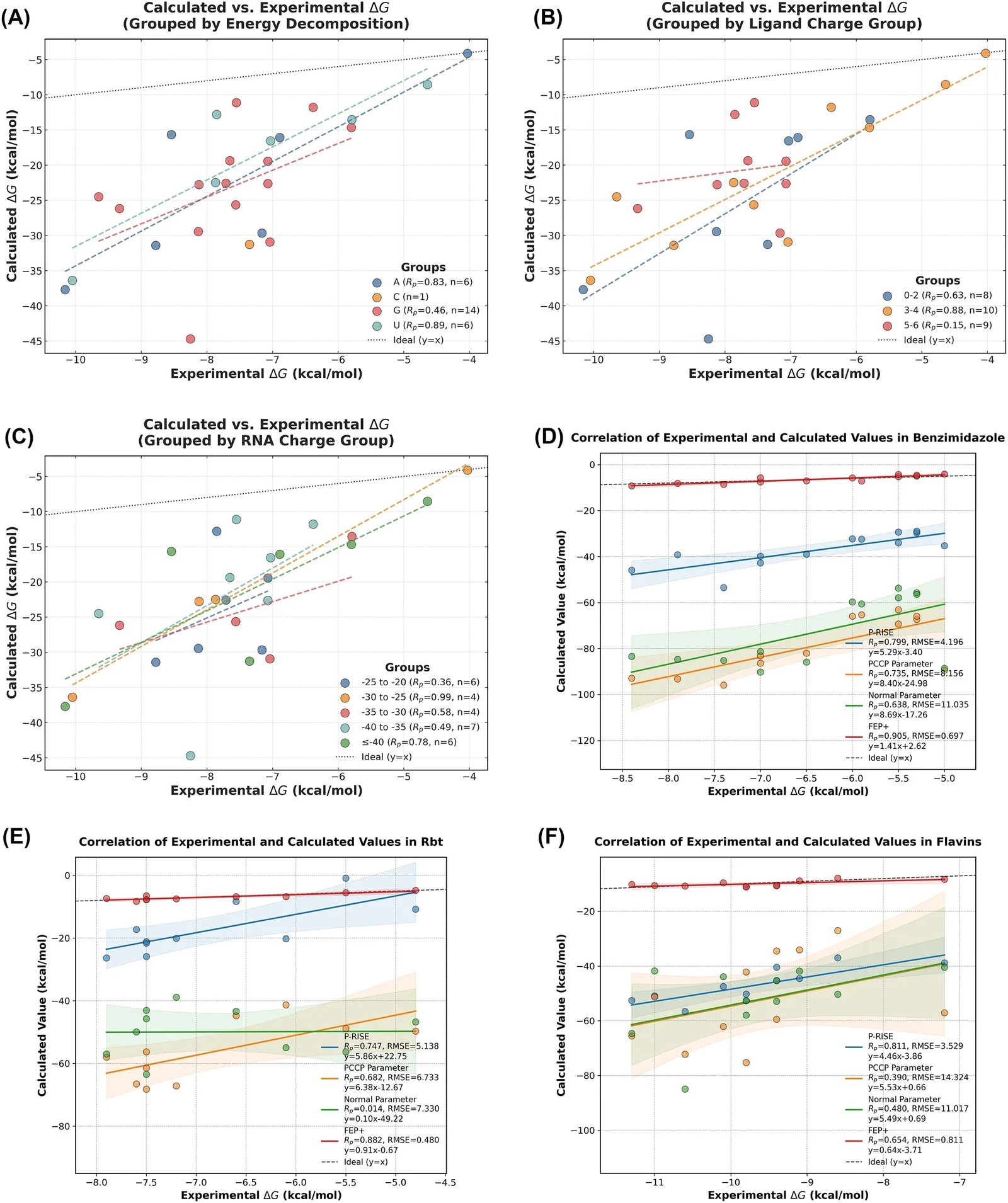
Benchmark analysis of P-RISE performance and applicability boundaries for RNA–ligand affinity prediction. (A–C) Method applicability boundaries based on system properties. (**A**) Performance comparison for systems dominated by the four nucleotides in the binding pocket. (**B** and **C**) Performance comparison for different ligand and RNA net charge, respectively. (D–F) Correlation between calculated and experimental binding free energies for benchmark Dataset II. The plots compare our P-RISE method (blue), the PCCP method (orange), and an unoptimized method (green). The performance of FEP+ is shown as a reference (red). The datasets are (**D**) Benzimidazole, (**E**) Rbt, and (**F**) Flavins.

Subsequently, we performed a similar classification based on the net charges of the small molecules and the RNA, again calculating *R*_p_ values for each subgroup. This analysis led to our third core finding: the existence of an “optimal charge window” associated with improved predictive accuracy. The highest performance is achieved when the ligand charge is approximately +3 to +4, and the RNA charge falls within a range of approximately −25 to −35 (Fig. 7B, C). This phenomenon reveals a fundamental applicability boundary for methods based on fixed-charge force fields. Within this window, electrostatic attraction is the primary driving force for binding, strong enough for its “signal” to dominate the computational “noise” from less accurate terms such as conformational entropy and solvation energy, thus ensuring robust predictions. However, when the system charge is excessively high, effects such as electronic polarization induced by the strong electric field and complex, competitive rearrangements of counterions at the binding interface become non-negligible. Fixed-charge force fields, incapable of describing these phenomena, systematically overestimate electrostatic attraction, leading to errors analogous to “electrostatic collapse”. Conversely, when the system charge is too low, the electrostatic driving force weakens, and the signal-to-noise ratio of the total binding energy decreases. Consequently, the calculation becomes hypersensitive to the inherent uncertainties in high-variance energy terms (especially conformational entropy and solvation free energy), where minor computational errors are amplified, ultimately leading to a loss of predictive power. This “optimal charge window” is therefore unlikely to be a purely incidental observation and is plausibly related to the trade-offs of fixed-charge force fields in balancing dominant electrostatics, polarization effects, and higher-order energy terms.

**External validation: performance comparison with benchmark methods**. To assess the generalization capability of the P-RISE framework, we benchmarked its performance on the binding free energy Dataset II against several methods: (i) the optimal protocol from a previous PCCP study (pure water, igb = 8, ε_in _= 4) [24]; (ii) a baseline endpoint method (pure water, igb = 5, default dielectrics); and (iii) the theoretically most rigorous free energy perturbation (FEP+) method. Predictive quality was judged by the *R*_p_ between calculated and experimental values. Dataset II comprises three subsets. The Benzimidazole and Rbt subsets contain ligands whose properties fall within the core application domain established in our study (Supplementary Tables S3 and S4). The third, the Flavins subset, constitutes a deliberate out-of-domain, adversarial test (Supplementary Table S5). Its ligand net charge transitions from −1 to 0, which lies not only outside the optimal charge window (+3 to +4) but entirely outside the 0–6 ligand charge range spanned by Dataset I (Fig. 7B), on which the applicability domain was characterized; it is moreover embedded in a strongly negatively charged RNA receptor (−109e). This subset was therefore chosen precisely because it breaks the assumptions of the method, rather than because it satisfies them. It provides a genuine extrapolation test of robustness and extensibility, and constitutes the primary basis for our claim of generalization beyond the calibrated domain.

The results demonstrate that, on chemotypes distinct from Dataset I yet within the pre-defined domain, the P-RISE framework performs excellently on both the Benzimidazole and Rbt datasets (Fig. 7D, E). The *R*_p_ values reach 0.799 and 0.747, respectively, approaching the performance of the gold standard FEP+ method (*R*_p_ = 0.905 and 0.882) while offering a profound advantage in computational cost. Whereas FEP+ requires 240–480 ns of total simulation time for a single relative free energy calculation, our method requires only 2–6 ns, representing an efficiency gain of nearly two orders of magnitude. This outstanding balance of accuracy and efficiency is precisely what is needed to address notoriously difficult challenges in RNA-targeted drug discovery, such as the high-throughput evaluation and prioritization of hits for cryptic binding sites that undergo induced-fit conformational changes, a problem recently highlighted by Olenginski *et al*. [67]. This outstanding balance between predictive accuracy and computational cost validates the practical value of our protocol’s construction and significantly outperforms all other endpoint methods tested.

The evidence for generalization beyond the calibrated domain, by contrast, rests on the out-of-domain Flavins subset. Critically, P-RISE retains predictive correlation on this adversarial case (*R*_p_ = 0.811), even exceeding the FEP+ result on the same benchmark (*R*_p_ = 0.654) (Fig. 7F), despite the ligand charge lying outside the entire charge range on which the domain was defined. This out-of-domain performance, rather than the within-domain reproducibility above, is what substantiates the extensibility of the method. One possible explanation is that the two methods respond differently to large charge changes and highly charged RNA environments. FEP+ relies on simulating an alchemical transformation pathway, where its soft core potential may face sampling convergence challenges when handling drastic charge changes (e.g. from −1 to 0). This challenge is likely to be exacerbated in the Flavins system, where the RNA receptor itself carries a strong negative charge of −109e, creating an extremely complex electrostatic environment. In contrast, our endpoint method is not path dependent and directly computes the energy difference between stable initial and final states. It may therefore be less sensitive to abrupt changes in ligand structure or charge in this particular system. Finally, while our method cannot match the absolute accuracy (RMSE) of FEP+ due to its theoretical approximations, it achieved the lowest RMSE among all endpoint methods compared. Taken together, these results show that P-RISE provides strong ranking performance within its current applicability domain, while maintaining substantially lower computational cost than alchemical approaches.

#### P-RISE improves pose discrimination relative to current RNA scoring and structure-generation approaches

Accurate prediction of the ligand binding pose is a prerequisite for reliable binding free energy calculations and a cornerstone of virtual screening. To systematically evaluate this capability, the P-RISE framework was first benchmarked against traditional physical scoring functions and a state-of-the-art statistical potential using Dataset III (Supplementary Table S7). We then compared P-RISE with representative existing approaches, including a statistical potential and AlphaFold3-derived [15] rankings, to examine how physics-based scoring and structure-generation models differ in RNA–ligand pose evaluation.

As shown in Fig. 8A, the full P-RISE scoring function (enthalpy + entropy) achieved the highest pose discrimination accuracy, with a Top-1 success rate of 75% under the present crystallographic benchmark, yielding the highest Top-1 success rate among the tested models. The enthalpy-only component of P-RISE ranked second, consistent with a contribution of high ionic strength modeling to stabilizing correct binding modes. Notably, P-RISE substantially surpassed AnnapuRNA (45% success rate), the leading knowledge-based statistical potential for RNA–ligand systems [23]. This performance gap suggests that statistical potentials may be less sensitive than P-RISE to the energetic distinctions required to rank near-native conformations in flexible RNA pockets.

**Figure 8.**
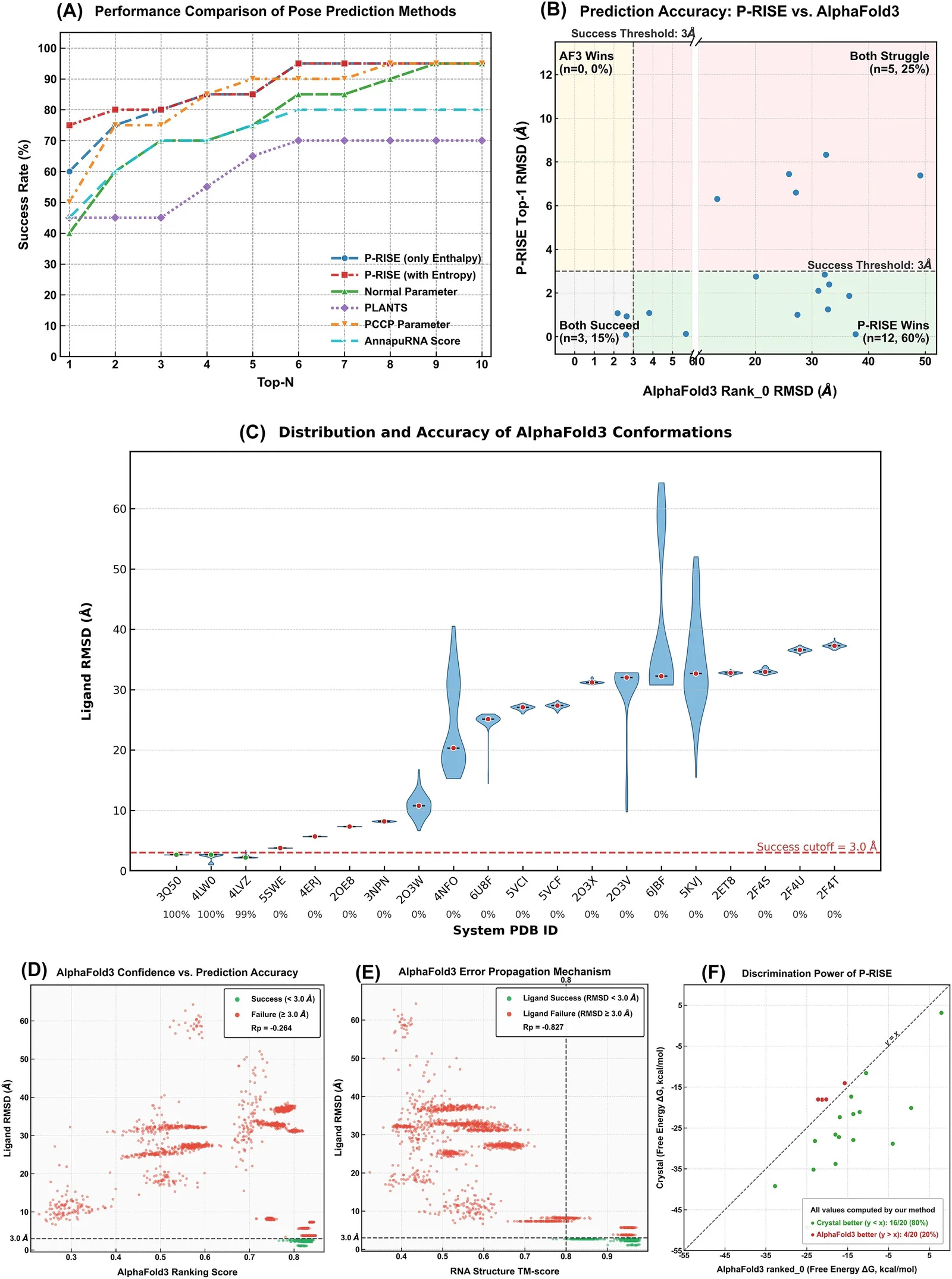
P-RISE improves RNA–ligand pose discrimination and provides complementary evaluation to AlphaFold3-derived predictions. (**A**) Comparison of Top-1 success rates for binding pose prediction on benchmark Dataset III. The methods compared include the P-RISE method (enthalpy + entropy), the method from the PCCP paper (igb = 8, ε_in_ = 4), the enthalpy-only component of P-RISE, the ‘normal’ parameters (igb = 5, default dielectric constant), the representative statistical potential-based scoring function AnnapuRNA score, and the PLANTS docking software. (**B**) Head-to-head comparison of prediction accuracy. Scatter plot comparing the ligand RMSD of P-RISE’s Top-1 prediction (*y*-axis) against AlphaFold3’s ranked_0 prediction (*x*-axis) across benchmark Dataset III. Dashed lines indicate the 3 Å success threshold and partition the plot into cases where both succeed, both fail, or one succeeds while the other fails (notably, the bottom-right quadrant indicates P–RISE success when AlphaFold3 fails). P-RISE achieves a 75% success rate versus AlphaFold3’s 15% and successfully predicts 70.6% of cases where AlphaFold3 fails. (**C**) Analysis of AlphaFold3’s conformational sampling. Violin plots showing the RMSD distribution of 100 poses per system from AlphaFold3. The uniformly narrow violins indicate low sampling diversity, while their position relative to the 3 Å threshold reveals a failure mode of being “collectively right or collectively wrong”. (**D**) Relationship between AlphaFold3 ranking_score and ligand RMSD. Scatter plot for all 2000 poses correlating AlphaFold3’s ranking_score with ligand RMSD. The disordered cloud and low *R*_p_ = 0.264 demonstrate that the internal score cannot reliably identify accurate poses. (**E**) Error propagation from RNA structure to ligand pose. Scatter plot of ligand RMSD versus the corresponding RNA TM-score. The analysis reveals a critical dependency: successful ligand predictions (green dots) only occur when the RNA structure is accurately predicted (TM-score >0.8), exposing an error propagation mechanism. (**F**) Validation of our P-RISE ’s discriminatory power. A “touchstone” test where our P-RISE evaluates AlphaFold3’s ranked_0 pose (*x*-axis) against the native structure (*y*-axis). With 80% of points below the *y* = *x* diagonal, the plot confirms P-RISE’s ability to robustly identify native-like conformations by assigning them more favorable scores.

Similarly, P-RISE outperformed the static energy minimization approach (PCCP). By relying on energy minimization of static structures, the PCCP method is prone to becoming trapped in local minima that appear energetically favorable but are structurally incorrect—a behavior consistent with the limitations of evaluating static snapshots without explicit dynamic sampling. In contrast, the P-RISE protocol explicitly incorporates system dynamics. By combining MD sampling with a comprehensive enthalpy–entropy scoring model, P-RISE identifies binding modes that are both energetically favorable and dynamically stable. This synergy allows the method to overcome the limitations of static physical models and the precision ceiling of statistical potentials.

To test whether restricting Dataset III to crystallographic structures biases the benchmark toward conformationally rigid systems, we constructed an ensemble-aware control set from 41 NMR-derived RNA–ligand complexes using an identical protocol (Supplementary Fig. S11). Because an NMR entry provides multiple equally valid deposited conformers, poses were generated by docking against all conformers and pooled, and performance was assessed using the minimum ligand RMSD over all conformers (best-match) as the primary criterion; this choice rests on the observation that the bias introduced by the equally defensible ensemble-average criterion is dictated almost entirely by the intrinsic dispersion of the experimental reference rather than by pose quality (Supplementary Fig. S11B). Under the best-match criterion, P-RISE reached a Top-1 success rate of 61.0%, with an improvement over the PLANTS scoring baseline of the same order as that obtained on the crystallographic set and Top-*N* curves of consistent shape (Supplementary Fig. S11A); as the two sets differ in the number of systems and in the origin of the receptor conformations, we compare only the improvement over the respective baselines and the overall trend, not absolute values. A per-system analysis showed that the controlled decoy set of every one of the 41 systems contains near-native poses, that the first near-native pose falls within the top three ranks for 33 systems, and that the few ranking failures are concentrated in systems with more solvent-exposed binding sites (Supplementary Fig. S11C). Finally, the Top-1 ligand RMSD showed no correlation with the ensemble divergence of each system (Supplementary Fig. S11D), indicating that pooling poses generated against different receptor conformers introduced no systematic bias coupled to conformational heterogeneity.

Given the rapid emergence of AI-based structure prediction, we further benchmarked P-RISE against AlphaFold3 to assess how its top-ranked predictions compare with those selected by a physics-based scoring framework. As AlphaFold3 is currently one of the few and most established end-to-end models capable of predicting RNA–ligand complexes, it was selected as the representative baseline. Recognizing the fundamental methodological difference between end-to-end prediction and physics-based scoring, we designed a comparative analysis to dissect the distinct advantages and limitations of each approach in the context of RNA–ligand recognition.

**Head-to-head comparison: top-tier accuracy of P-RISE versus AlphaFold3**. To first directly compare the final prediction accuracy, we benchmarked our Top-1 prediction (i.e. the highest-ranked pose) against AlphaFold3’s ranked_0 prediction (i.e. its highest-confidence pose). The results are presented in the 2D scatter plot in Fig. 8B.

The results reveal two consistent trends. First, AlphaFold3 achieved success in only 15% of the systems, whereas the Top-1 success rate of our method reached 75%. This indicates that, under the present benchmark conditions, P-RISE more frequently identified near-native poses than AlphaFold3 ranked_0 predictions. Second, the top-left quadrant (P-RISE fails/AlphaFold3 succeeds) is unpopulated, indicating no systems where AlphaFold3 succeeded while our method failed. In stark contrast, the bottom-right quadrant (P-RISE succeeds/AlphaFold3 fails) is densely populated. This quadrant contains 12 systems, meaning that of the 17 cases where AlphaFold3’s prediction failed (RMSD >3 Å), our method successfully predicted the correct pose for 12 of them—a success rate of 70.6% on these challenging targets. These results indicate that P-RISE provides more consistent pose selection than AlphaFold3 in this benchmark, particularly for systems that were not correctly ranked by AlphaFold3.

**In-depth diagnosis: intrinsic mechanisms of AlphaFold3’s predictive failures**. Having established the overall superiority of our method, we proceeded to investigate the underlying reasons for AlphaFold3’s predictive failures. Given that AlphaFold3 is an end-to-end model, whereas our P-RISE protocol evaluates poses generated via traditional docking, we first sought to determine if inadequate sampling by AlphaFold3 was the primary cause of its inferior performance. We analyzed the distribution and diversity of the 100 candidate poses generated by AlphaFold3 for each system, as depicted in Fig. 8C.

A clear observation is that the “violins” for most systems are extremely narrow, indicating a lack of structural diversity among the generated poses. More importantly, the distribution of these violin plots reveals a stark “all-or-nothing” pattern. For systems 3Q50, 4LW0, and 4LVZ, the violins are located entirely below the success threshold of *y* = 3 Å, showing that all predictions are clustered around the correct conformation. For the remaining systems, however, the violins are suspended far above the 3 Å threshold, meaning that all predictions are densely clustered in an incorrect region, far from the native pose. This finding suggests a limitation of AlphaFold3 sampling diversity under the conditions tested here: its conformation generation process, which is primarily driven by a regression model, appears to explore a relatively narrow region of conformational space in these systems. Consequently, if the regression model encounters an unfamiliar system and its initial prediction deviates from the correct solution, its limited sampling and refinement steps are insufficient to escape this local minimum, resulting in a “collective failure” of the entire output pose pool.

Given the lack of diversity in AlphaFold3’s conformational sampling pool, we next asked whether its built-in confidence score (ranking_score) possesses discriminatory power. If the scoring were reliable, it should at least be able to select the best pose from the generated pool. To test this hypothesis, we generated the scatter plot in Fig. 8D, which includes all 2000 predicted poses (20 systems × 100 poses). Our objective was to determine if a negative correlation exists, where a higher score corresponds to a lower RMSD.

However, the plot reveals an amorphous cloud of data points with no discernible trend. To quantify this, we calculated the Pearson correlation coefficient (*R*_p_), which yielded an absolute value of merely 0.264, confirming the absence of a meaningful linear relationship. This result indicates that AlphaFold3’s internal confidence score is only weakly associated with pose accuracy in this RNA–ligand dataset. This observed decoupling between the AI-predicted ranking and physical reality is not an isolated issue; it echoes recent critical evaluations in the protein–ligand field, where it was shown that even state-of-the-art co-folding models often fail to capture the underlying physics of biomolecular interactions [68]. Our analysis indicates that this limitation, previously reported in the protein–ligand field, is also evident in our RNA–ligand benchmark. A high confidence score does not equate to a low RMSD, indicating that AlphaFold3’s built-in scoring module was not, under the conditions tested here, sufficiently reliable for prioritizing correct poses in this RNA–ligand benchmark. These observations suggest that current AI-based methods such as AlphaFold3 are not yet optimized for reliable RNA–ligand energetic ranking, and that energy-based evaluation provides substantial complementary value in this benchmark, supporting the rationale of our approach.

After evaluating conformational sampling and confidence scoring, we investigated a deeper potential cause: whether AlphaFold3’s performance is constrained by its ability to predict the RNA receptor structure itself. In other words, is the inaccuracy in RNA–ligand prediction a downstream consequence of inaccuracies in the RNA structure prediction? To explore this, we plotted Fig. 8E, correlating the TM-score of the RNA component (a measure of backbone topological accuracy relative to the crystal structure) with the RMSD of the ligand.

The result in Fig. 8E presents a distinct “L-shaped” or right-angle distribution. By drawing dashed lines at *x* = 0.8 (TM-score) and *y* = 3 Å (RMSD), we partitioned the plot into four quadrants. A decisive observation emerged: nearly all successful predictions (green points, RMSD <3 Å) are exclusively clustered in the bottom-right quadrant, a region characterized by very high RNA TM-scores (>0.8). Conversely, in the region where the RNA TM-score is below 0.8, successful ligand pose predictions are virtually non-existent. This reveals a critical error propagation mechanism: the success of AlphaFold3’s ligand pose prediction is strongly dependent on its accurate reconstruction of the RNA receptor structure. When RNA backbone accuracy decreases, ligand pose accuracy also tends to deteriorate. Under the conditions tested here, this suggests that current co-folding architectures face difficulty in jointly handling the coupled problem of receptor–ligand co-folding for flexible RNA systems, where errors in receptor reconstruction tend to propagate to the ligand pose.

**Synergy and transcendence: the value of P-RISE as a physical validation layer**. Our comprehensive analysis thus far has clearly identified the multiple limitations of AlphaFold3 in conformational sampling, confidence scoring, and receptor flexibility handling. This, however, raises a critical question: is the performance advantage of our method demonstrated in Fig. 8B attributable to superior conformational sampling, or to the intrinsic discriminatory power of its underlying energy model?

Answering this question is important because P-RISE is, at its core, an energy evaluation framework whose central innovation lies in “evaluation” rather than “sampling”. The head-to-head comparison in Fig. 8B evaluates two coupled, end-to-end workflows, making it impossible to isolate the contribution of the energy model alone. Therefore, to truly test the value of our method, it is essential to decouple it from the sampling step (i.e. docking) and evaluate its intrinsic discriminatory power in isolation. To this end, we designed a rigorous evaluation: we used the P-RISE energy model to evaluate two distinct structures: the AlphaFold3-predicted ranked_0 pose (which is typically incorrect) and the native crystal structure (the “ground truth”). If our model is reliable, it should consistently assign a more favorable (i.e. lower) energy value to the “ground truth”.

The results of this experiment are shown in Fig. 8F. In this plot, a clear majority of the data points (16 out of 20, or 80%) fall below the *y* = *x* diagonal. This demonstrates that for nearly all systems, our energy model assigned a lower (more favorable) energy value to the native conformation than to the incorrect pose predicted by AlphaFold3. This result supports the view that the energy evaluation component of P-RISE contributes substantially to its overall success in pose discrimination. It acts as an effective filter that, in this benchmark, distinguishes “true” native-like poses from “false” incorrect ones. It is this reliable discriminatory ability that gives our method the potential to accurately identify and select the near-native pose from any conformational ensemble, including those of low quality generated by AlphaFold3.

Synthesizing the comprehensive comparisons against both traditional methods and AI co-folding models, we arrive at the fourth core finding of this study: in the task of RNA–ligand complex structure prediction, current AI models explore chemical space broadly but assess physical plausibility less reliably; a physics-based energy model can help bridge the two. AI co-folding models such as AlphaFold3 can be viewed as powerful “conformational hypothesis generators”, but they show limitations in sampling diversity and self-assessment. The core value of P-RISE, in contrast, lies in its function as a “physical plausibility filter”, which, by leveraging its reliable energy discrimination, can often identify the correct, near-native conformation from within a conformational ensemble, even one of low quality. This reveals that physics-based energy methods are not obsolete; on the contrary, they play a valuable complementary role in the AI era as a physics-based validation layer, offering the necessary physical constraints to ground data-driven predictions in energetic reality.

### Physical priors remain advantageous under current RNA data constraints

It was established above that even state–of–the–art co–folding models such as AlphaFold3 require an explicit physical discrimination layer to validate RNA–ligand complex predictions. This observation naturally raises a broader methodological question relevant to practical drug discovery: in the data–poor regime characteristic of RNA–ligand systems, can data–driven models still improve predictive performance through increased model complexity and enriched feature representations, as observed in protein systems?

To address this question under controlled conditions, we established a systematic comparative framework tailored for small-sample regimes, designed to explicitly distinguish between information derived solely from data and information introduced by physical priors. Specifically, we contrasted two strategies: (i) Pure-AI, which relies exclusively on conventional physicochemical descriptors; and (ii) Phys-AI, which augments these descriptors with physical and dynamic features derived from P-RISE/MD simulations. This experimental design enabled us to examine whether physics-guided feature augmentation could yield consistent, additive performance gains.

Within this framework, we enforced a unified protocol for model selection and hyperparameter optimization and evaluated performance across multiple dimensions, including in-sample fitting, external transferability to independent datasets, and—where applicable—structural fidelity. Concurrently, we employed diagnostic tools, such as learning curves and feature attribution, to quantify the constraints imposed by data scarcity on learnability and to elucidate the impact of physical priors on the boundaries of attainable performance. The details of feature space construction, modeling protocols, and result analyses are presented in the Materials and methods.

#### Feature space construction and robust modeling strategy

To establish an interpretable and reproducible comparative framework, we first constructed a feature space encompassing multiple sources of information. A broad set of conventional physicochemical descriptors was computed using RDKit and MOE. In parallel, high-dimensional dynamic features were extracted from P–RISE simulations using the AmberTools suite, including P–RISE energy terms as well as trajectory-based metrics such as RMSD and RMSF. This procedure resulted in an initial feature set comprising 146 dimensions.

To ensure both input orthogonality and physical interpretability, we applied stringent variance filtering and collinearity reduction. Within strongly correlated feature groups, the feature with the clearest physical meaning was deliberately retained. After this curation process, two core feature sets were defined. The first consisted of 21 dimensions of purely physicochemical descriptors and was designated as Pure AI. The second extended this set by incorporating 11 MD/P–RISE–derived features, yielding a 32–dimensional hybrid representation referred to as Phys–AI.

It is worth noting that an ML model trained exclusively on physical features (Physics–only ML) was intentionally excluded from the experimental design. Rather than constituting an independent predictive paradigm, fitting a statistical model solely to P–RISE energy terms would merely serve as a noisy, regressive approximation of the underlying physical Hamiltonian. In the data–poor regime, such an approach is unlikely to introduce additional epistemic gain beyond established physical laws. Instead, it risks amplifying statistical noise through overparameterization, ultimately degrading the fidelity of physical inference. Therefore, our analysis focuses on the performance boundary between data mining without physical priors (Pure AI) and physics–guided hybrid augmentation (Phys–AI).

With the feature space defined, model selection became the subsequent critical control point. Given the extreme data scarcity (*N* = 27 samples) relative to feature dimensionality, we adopted a deliberately conservative strategy to prevent overfitting. We excluded deep learning architectures (e.g. GNNs and Transformers) and sparse binary fingerprints, which typically require large datasets to converge. Instead, we selected a diverse set of classical algorithms—XGBoost (XGB), Random Forest (RF), Gaussian Process Regression (GPR), Support Vector Machines (SVM), and a shallow Multi-Layer Perceptron (MLP)—to enable a cross-paradigm assessment.

To minimize false negatives arising from insufficient tuning and false positives driven by overfitting, we employed a conservative hyperparameter optimization protocol. The parallel–coordinate plots in Supplementary Figure S12 illustrate the exploration of this high–dimensional parameter space. Given that the number of features slightly exceeds the number of samples, grid search was not designed to maximize training performance alone. Instead, it explicitly balanced training accuracy with generalization performance assessed by LOOCV. This procedure ensured that each algorithm converged toward its most robust operating regime rather than its most optimistic fit.

Finally, following the “garbage–in, garbage–out” principle, only P–RISE energy terms that had undergone explicit parameter optimization were used as physical inputs. Standard implicit–solvent model parameters not optimized for RNA systems were intentionally excluded. This ensured that the learning models were trained on high–quality thermodynamic signals rather than noisy or physically inconsistent inputs.

#### Result analysis: physical priors improve learning but explicit physics remains a stronger baseline

Within the unified framework described above, we first evaluated in–sample performance on Dataset I, a heterogeneous absolute binding free–energy (ABFE) dataset. The performance trajectories in Fig. 9A reveal a clear stratification in data efficiency. In the Pure–AI regime on the left, where only 21 descriptor–based features are used, predictive performance remains limited by the small sample size. When the feature space is expanded to include 11 P–RISE/MD–derived physical features, all five learning algorithms exhibit a sharp improvement in performance. Across both feature settings, the MLP model achieves the highest prediction accuracy, benefiting from its non-linear fitting capacity. Nevertheless, a critical observation emerges. Even the best–performing Phys–MLP model does not reach the performance level of the purely physics–based P–RISE method, indicated by the gold dashed line in Fig. 9A (*R*_p_ = 0.696). This result explains why the final methodological selection favors the physical approach. Despite integrating AI throughout the pipeline and providing it with the strongest available physical features, the hybrid models do not surpass the explicit physical formulation. At the current data scale, the experimental data simply do not contain sufficient information to correct or refine the underlying physical laws, leaving the physics–based method as the more reliable reference.

**Figure 9.**
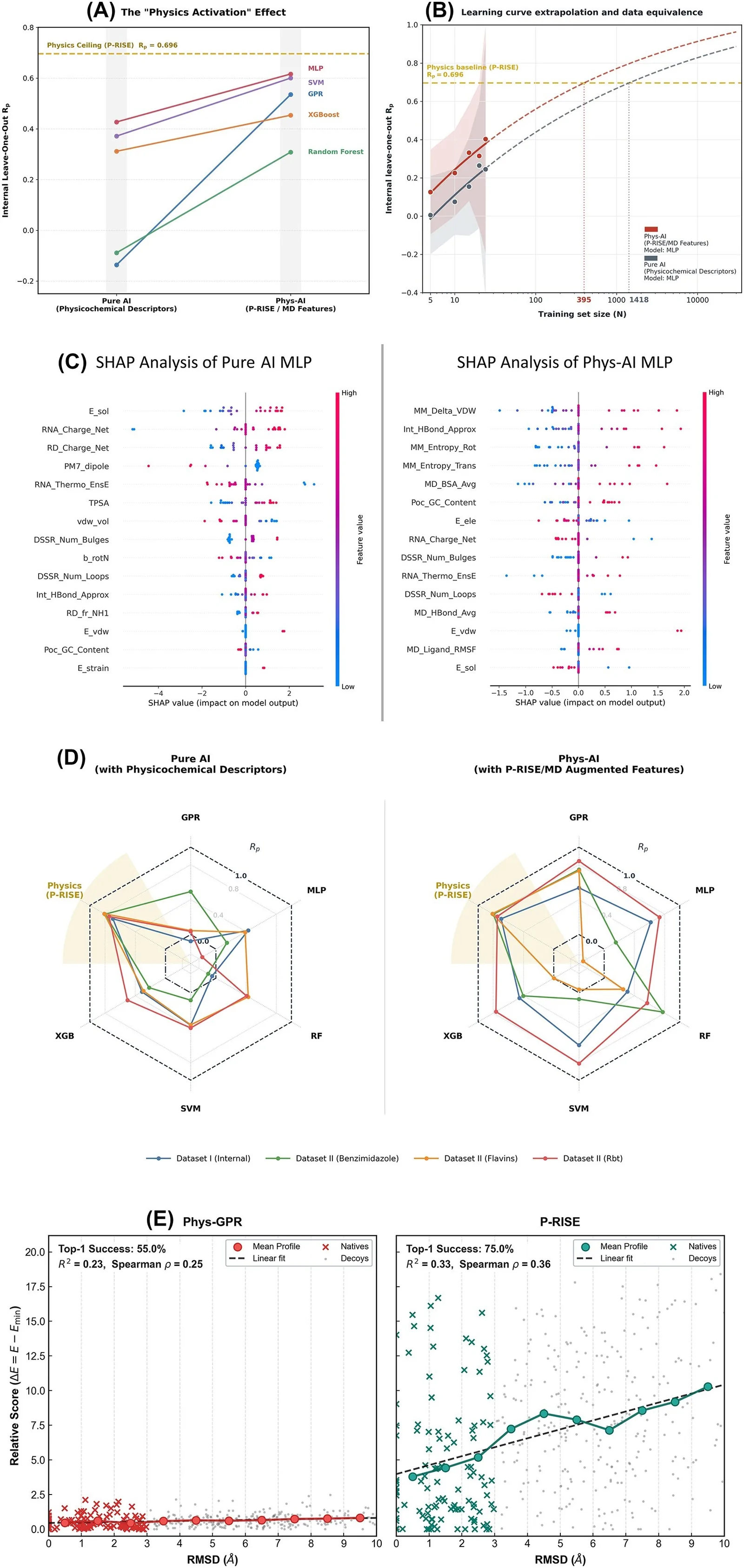
Effects of physical feature augmentation on ML performance and transferability. (**A**) Physics activation effect on AI predictive capacity. Performance trajectories of five ML algorithms (GPR, SVM, RF, XGB, and MLP) when transitioning from pure physicochemical descriptors (left) to physics-augmented features incorporating P-RISE/MD information (right). Internal leave-one-out *R*_p_ values show consistent improvement across all methods, though remaining below the physics-based ceiling (P-RISE, dashed line, *R*_p_ = 0.696). Vertical bands denote feature space domains. (**B**) Data efficiency comparison. Learning curves (*R*_p_ versus *N*) for Pure AI (gray) and Phys-AI (red). Solid and dashed lines denote experimental data (*N* = 5–24, mean ± SD) and inverse power law extrapolations [*R*_p_**(N)** = L − a*N*^−b^], respectively. Vertical dotted lines mark the sample sizes required to match the physics baseline (*R*_p_ = 0.696, gold): Phys-AI achieves parity at *N* ~395, whereas Pure AI requires *N* ~1418, indicating a 3.6-fold reduction in data requirements via physical priors. (**C**) Feature attribution and physical term dominance. SHAP summary plots displaying the top 15 features for Pure AI (left) and Phys-AI (right). While Pure AI relies on physicochemical properties such as solvation and charge, the Phys-AI model is strictly dominated by P-RISE and MD-derived terms. This prioritization indicates that the performance gain stems from “physics recovery”—essentially performing weighted regression on known physical laws—rather than the emergence of novel chemical insights. (**D**) Contrast in generalization stability between physics-based and data-driven models. Pure AI models (left) exhibit severe instability and generalization failure on external datasets (Dataset II). While integrating physical features (right) yields marginal recovery (notably in GPR), most ML models still fall short of the robustness exhibited by the pure physics approach. In contrast, the P-RISE method (highlighted yellow axis) maintains consistently high *R*_p_ levels across all four systems. This consistency is in line with a physics-based origin of the stability, which data-driven models struggle to replicate even with feature augmentation; we interpret it as evidence of predictive robustness rather than as direct proof that each physical term is individually correct. (**E**) Pose-level scoring behavior of Phys-GPR and P-RISE. Scatter plots show system-wise relative energies, Δ*E* = *E* − *E*_min_, versus ligand RMSD for Phys-GPR (left) and P-RISE (right). Relative energies retain units of kcal mol^−1^ and remove only the system-specific energy offset, thereby preserving pose-dependent energetic differences within each scoring method. Gray dots represent decoy poses, whereas colored crosses indicate near-native poses (RMSD <3 Å). Solid lines with circles represent mean relative energy profiles averaged over 1 Å RMSD bins, and black dashed lines show global linear regressions. The strength of the score–RMSD association is summarized by *R*^2^ and Spearman ρ, while practical pose discrimination is evaluated by the Top-1 success rate. Phys-GPR retains a limited pose-related trend but compresses candidate poses into a narrow predicted energy interval. P-RISE preserves broader pose-dependent energetic contrast, shows a stronger score–RMSD association, and achieves a higher Top-1 success rate (75% versus 55%).

Learning curve analysis further quantifies the value of this physical prior (Fig. 9B). The limited data range explored here corresponds to a pronounced high–variance valley. The Pure–AI curve (gray solid line) is accompanied by an exceptionally wide uncertainty band, indicating substantial predictive instability. The Phys–AI curve (red solid line) shows higher mean accuracy, yet its uncertainty does not narrow accordingly. In some regions, its SD even exceeds that observed at the *N* = 5 baseline. This behavior reflects an increase in model degrees of freedom introduced by high–dimensional physical features, which amplifies variance when data are insufficient and leads to highly uncertain predictions. In contrast, the physics–based P–RISE method, being parameter free, entirely bypasses this instability and appears as a stable gold baseline. Quantitative extrapolation (dashed lines) using an inverse power law fit indicates that reaching the P–RISE performance level would require ~395 samples for Phys–AI models and ~1418 samples for Pure–AI models. This large horizontal gap implies that the physical prior embedded in P–RISE is, from an information–theoretic perspective, equivalent to saving ~400–1400 expensive experimental measurements.

To further interrogate the origin of the performance gain observed for Phys–AI over Pure-AI, we analyzed feature attributions using SHAP, as shown in the bee–swarm plots in Fig. 9C. The left panel corresponds to the Pure–AI models. Here, solvation energy emerges as the most influential feature, followed by the charge properties of the ligand and RNA. This ranking carries clear scientific significance. It not only confirms the central role of solvation effects in RNA–ligand binding, but also directly validates the rationale of our earlier domain applicability partitioning of P–RISE based on charge characteristics. In contrast, the right panel of Fig. 9C reveals a markedly different attribution pattern for the Phys–AI models. The top–ranked features are dominated almost entirely by P–RISE– and MD–derived terms. This indicates that, at the present data scale, ML does not acquire emergent chemical insight that is independent of the physical model. Instead, it effectively reduces to a linear reweighting of existing physical terms, amounting to a form of physical reproduction rather than genuine knowledge discovery. From a methodological perspective, this behavior exposes the cognitive ceiling of data–driven approaches operating in a data–poor regime.

High correlations in scalar binding energies do not necessarily guarantee equally effective pose ranking. To examine whether the hybrid model retained pose-related scoring information, we selected Phys-GPR, which showed the most robust transfer behavior among the tested learning models, and compared its conformational scoring performance with that of P-RISE on the same candidate poses (Fig. 9E). For both methods, the plotted quantity is the system-wise relative energy, Δ*E* = *E* − *E*_min_, expressed in kcal mol^−1^. This representation removes the arbitrary offset of each receptor–ligand system while retaining the pose-dependent energetic differences predicted by each scoring scheme.

We further observed that this strong reliance on physical features leads to pronounced instability during cross–system generalization. When the trained models were evaluated on the fully independent Dataset II, an RBFE dataset comprising three congeneric series, the radar plots in Fig. 9D reveal two sharply contrasting patterns. Crucially, on both graphs, all the curves along the P–RISE axis remain consistently high, exhibiting pronounced invariance across datasets. This pattern indicates that the explicit physics-based framework transfers more consistently than the fitted models across the datasets examined here. In contrast, the Pure–AI models appear as collapsed, highly distorted polygons with negligible area, indicating a near-total failure under external validation. While the Phys–AI models expand the coverage area, they introduce significant variance. The MLP axis shows high dispersion, reflecting strong training performance but unstable generalization; conversely, the GPR axis shows tighter clustering, suggesting that kernel-based interpolation offers superior robustness in this regime. Notably, the radar plots also reveal a seemingly counterintuitive phenomenon. In some cases, validation performance on Dataset II (RBFE) even exceeds training performance on Dataset I (ABFE). We emphasize that this does not indicate improved model capability. Rather, it reflects the intrinsic physical nature of RBFE tasks, where systematic errors—such as receptor strain and conformational entropy—tend to cancel among congeneric compounds, thereby reducing predictive difficulty.

Under this representation, both methods showed increasing relative energy with increasing RMSD, indicating that both retain some pose-related information. However, the score–RMSD association was stronger for P-RISE than for Phys-GPR, as reflected by both the coefficient of determination and the rank-based correlation (*R*^2^ = 0.33 and ρ = 0.36 for P-RISE; *R*^2^ = 0.23 and ρ = 0.25 for Phys-GPR). We therefore no longer interpret the raw visual steepness of the two curves, or the slope of the global regression line, as scale-independent evidence of different funnel quality.

The narrower relative energy range observed for Phys-GPR is not merely a plotting artifact but reflects compression of pose-dependent score differences by the regression model. Phys-GPR was trained on experimental binding free energies spanning −4.3 to −10.16 kcal mol^−1^, and after per-system referencing only within-system pose variation remains. Because the model is optimized primarily for cross-system affinity prediction and, as a conditional mean regressor, tends to shrink predictions toward the label mean, it yields a reduced dynamic range at the pose level. Consistent with this compression, P-RISE achieved a higher Top-1 pose selection success rate than Phys-GPR (75% versus 55%). Thus, rather than implying a complete loss of structural information in Phys-GPR, these results indicate that the hybrid model retains partial pose-related information but is less effective at preserving the energetic contrast required for near-native pose selection in this benchmark.

Taken together, the analyses spanning data efficiency (Fig. 9A/B), feature attribution (Fig. 9C), generalization behavior (Fig. 9D), and pose-level scoring (Fig. 9E) support the fifth core finding of this study. Under the present data-limited RNA–ligand setting, physical feature augmentation improves the performance of data-driven models but does not consistently exceed the P-RISE reference across the evaluated tasks. Phys-GPR retains partial pose-related information, whereas its regression output compresses pose-level energetic differences relative to P-RISE. Thus, within the present benchmark, explicit physical scoring provides a more effective and interpretable reference for preserving energetic contrast among candidate poses and selecting near-native conformations.

## Discussion

This work addresses a central challenge in RNA-targeted discovery: how to obtain reliable and transferable RNA–ligand predictions under conditions of limited structural data and pronounced electrostatic complexity. By systematically dissecting endpoint free energy calculations in RNA systems, we identify a set of coupled physical determinants that govern predictive stability and integrate them into the P-RISE framework. Across affinity ranking, ion site localization, and pose discrimination tasks, these analyses show that a physically grounded strategy can provide robust performance and clear interpretability under current RNA data constraints. Rather than positioning physics-based and data-driven methods as mutually exclusive paradigms, our results help clarify their respective roles in present-day RNA–ligand modeling.

We further note a limitation of the present validation. Although the out-of-domain Flavins subset provides a stringent extrapolation test, generalizability here is assessed through a single such subset. This reflects an objective bottleneck of the field—experimentally characterized RNA–ligand complexes with reliable binding affinities remain extremely scarce, which currently bounds the size and chemical diversity of any external RNA benchmark. We have therefore limited our generalizability claims to what the available data support, and regard the construction of larger, chemically diverse external RNA datasets, together with correspondingly stronger out-of-domain evaluation, as an essential direction for future work as such data become available.

A second, related limitation concerns the construction of the pose prediction benchmark itself. RMSD-based evaluation of pose prediction requires a single, unambiguously defined reference coordinate set, and this technical requirement makes Dataset III predominantly crystallographic, leaving it under-representative of highly flexible complexes characterized exclusively by NMR and potentially biasing the benchmark toward conformationally more rigid systems. We emphasize that RNA flexibility and dynamic ensembles are represented in this work through explicit MD sampling performed for every system rather than through experimentally deposited conformer sets, so this choice does not extend to our physical analyses of conformational dynamics and ion sensitivity. We examined the limitation further using an ensemble-aware control set of 41 NMR complexes: the improvement of P-RISE over the docking score baseline is of the same order as on the crystallographic set, the Top-*N* trends are consistent, and the Top-1 error is uncorrelated with the ensemble divergence of each system, indicating that discriminative performance does not depend on conformational rigidity. At the same time, this control set exposes an intrinsic metrological difficulty of multi-conformer references: two equally defensible RMSD criteria differ by 17.1% in Top-1 success rate, and one of them is mechanically coupled to the dispersion of the experimental reference itself. It is on this basis that we retain the crystallographically defined benchmark for quantitative evaluation while using the NMR set as an independent robustness check; developing ensemble-aware evaluation metrics that do not rely on a single reference conformer is a necessary direction for future RNA–ligand pose prediction benchmarking.

A further consideration concerns the statistical power of the core affinity benchmark, which comprises 27 systems—a size that warrants caution in interpreting absolute correlation coefficients. To guard against conclusions being driven by the particular composition of this set, we based our mechanistic claims not on absolute *R*_p_ values alone but on paired comparisons in which each system serves as its own control, so that between-system variance largely cancels. Under this analysis (Supplementary Table S6), every determinant retained in P-RISE produces a positive paired Δ*R*_p_. The two dominant contributions—the internal dielectric treatment [Δ*R*_p_ = 0.49, 95% CI (0.08–0.88)] and the choice of production window [Δ*R*_p_ = 0.19, 95% CI (0.06–0.30)]—are individually resolved at the present sample size, with their bootstrap CIs excluding zero. The ionic strength contribution carries a large point estimate (Δ*R*_p_ = 0.39) whose interval only marginally includes zero [−0.05 to 0.59], so its effect is sizeable but not statistically resolved at *N* = 27, while the entropy correction and the external dielectric treatment are directionally positive but likewise unresolved [Δ*R*_p_ = 0.07 (−0.05 to 0.22) and 0.005 (−0.004 to 0.013), respectively], consistent with their intended role as bounded relative ranking corrections rather than dominant determinants of *R*_p_. A leave-one-out test further confirms that the overall correlation is not contingent on any single complex (seed-averaged full-set *R*_p_ = 0.631; range 0.577–0.670 across removals; Supplementary Fig. S9G).

First, we establish P-RISE as a practical computational reference that balances efficiency with physical fidelity, while helping define its domain of applicability. Through rigorous validation on diverse datasets, we demonstrate that a protocol refined at the level of physical mechanisms can serve as a reliable source of ground truth. The core strength of this protocol lies in its physically consistent representation of the microscopic physical environment. Molecular mechanics are described using the OL3 force field, while simulations are conducted under elevated ionic strength conditions (e.g. 0.5 M MgCl_2_) that act as an effective electrostatic scaffold to stabilize the RNA tertiary fold and, critically, the local binding pocket. Although these bulk concentrations exceed physiological ionic strength, the local ionic environment around the polyanionic RNA backbone is itself far more concentrated than bulk values [63]; we therefore interpret this condition as a physically grounded stabilization strategy rather than as a literal reproduction of cellular electrolyte concentrations. A relatively high internal dielectric constant (ε_in_ = 20) is employed, and short but carefully selected 2 ns MD windows are used to balance sampling efficiency and structural stability—a choice that additionally confers a practical throughput advantage for large-scale screening. In the final energy evaluation, quasi-harmonic entropy corrections are combined with an ion-dependent external dielectric constant (ε_out_) to improve cross-system ranking. We note that, on nanosecond-scale trajectories of highly flexible RNA, these endpoint entropy terms cannot capture slow collective motions and are therefore employed as approximate relative corrections rather than converged absolute quantities; accordingly, the entropy–solvent coupling is best regarded as an empirically observed regularity rather than a fully resolved physical mechanism. This framework exhibits robust performance across ion site localization, external affinity evaluation, and pose discrimination tasks. More importantly, its construction on an explicit physical foundation endows P–RISE with a high degree of interpretability. This allows us not only to assess what the method can achieve, but also to delineate, on physical grounds, where it applies. We observe systematic fluctuations in prediction accuracy for systems dominated by guanine–centered recognition motifs or lying outside the optimal charge window. These cases do not reflect random algorithmic failure. Instead, they expose the limitations of current fixed–charge force fields in describing complex electronic effects such as polarization. This ability to identify and rationalize “difficult systems” reinforces the conclusion that P–RISE derives its performance from proximity to physical reality rather than from overfitting to specific datasets, supporting its use as a physically interpretable reference for current RNA–ligand prediction tasks.

Second, our analysis clarifies why the framework performs well in RNA systems. High ionic strength primarily stabilizes the RNA tertiary fold and, more importantly, preserves the integrity of the local binding pocket required for reliable energy evaluation. In parallel, the predictive accuracy of the free energy estimates appears to arise not from exact treatment of any single energetic term, but from an effective and physically interpretable error cancellation regime across electrostatics, solvation, and entropy. Under the current precision limits of fixed charge force fields, these observations suggest that self-consistency of the overall physical model may be more important than attempting to optimize individual terms in isolation.

This error cancellation perspective, however, also delineates the boundaries within which our conclusions should be read. Because the accuracy we report emerges from a favorable cancellation of systematic errors rather than from an exact treatment of any single term, it is subject to the intrinsic limitations of endpoint free energy methods for polyanionic RNA. The implicit solvent (GB) description of a highly charged backbone remains imperfect; the electrostatic cancellation on which the protocol relies is system dependent and is not guaranteed to transfer to markedly different receptors; the ion atmosphere is only partially represented by a mean-field, concentration-dependent dielectric; and the conformational entropy, even under the quasi-harmonic approximation, remains highly approximate. For this reason we interpret the improvements reported here as gains in the consistency and transferability of relative affinity ranking, and not as evidence that P-RISE recovers the true, mechanistically resolved energetics of RNA–ligand binding. In this sense, the contribution of P-RISE is one of predictive utility, a reproducible ranking reference under present-day data scarcity, whereas physically rigorous estimation of absolute RNA–ligand free energies remains an open problem beyond the validated scope of any current endpoint method. It is precisely this reliable, physics-grounded ranking behavior, rather than term-by-term physical exactness, that we believe qualifies P-RISE as a reference for the data-driven developments discussed next.

Underlying these endpoint-level limitations are the well-documented constraints of the classical force fields used for RNA simulation, which we make explicit here. The energetics reported in this work rest on a fixed-charge, non-polarizable representation: electronic polarization and charge transfer, both non-negligible around the densely charged phosphate backbone and at ion-binding sites, are captured only in a mean, pre-parameterized sense, which is a primary origin of the systematic deviations we observe for guanine-centered recognition and for ligands outside the calibrated charge window. Beyond the charge model, current RNA force fields carry known residual biases in backbone (α/γ) and glycosidic (χ) torsional preferences—torsions that have required successive quantum chemistry-based refinements [40, 41]—as well as in sugar pucker equilibria and in the description of non-canonical pairs and single-stranded/loop flexibility, where even state-of-the-art fields such as OL3 show documented strengths and weaknesses [39]; the resulting conformational ensembles are moreover sensitive to the specific force field/solvent model combination [38]. These are compounded by approximations in the surrounding environment model: fixed point charge water (e.g. TIP3P) imperfectly reproduces the dielectric and hydrogen-bonding behavior of the first hydration shell of a polyanion, and standard monovalent and, especially, divalent (Mg^2+^) ion parameters are known to imperfectly reproduce ion–phosphate interaction free energies and residence kinetics. We emphasize that these limitations are not incidental to our findings but are, in fact, the physical origin of the corrective strategies characterized in this study—the elevated internal dielectric, the ion-dependent external dielectric, and the bounded simulation window all act, in part, to counteract the accumulation of fixed charge force field error. Recognizing this explicitly reinforces our central positioning: P-RISE extracts stable, transferable ranking behavior from imperfect physical models, rather than claiming force-field-level mechanistic exactness, and it also identifies the improvement of RNA force fields, ultimately through polarizable or ML potentials, as the most direct route to systematically reducing these errors.

Third, our comparative analyses highlight the current value of physical priors under RNA data-limited conditions. In pose discrimination benchmarks, P-RISE improves upon both statistical potentials and AlphaFold3-derived top-ranked predictions, indicating that explicit energetic evaluation remains highly informative for RNA–ligand modeling. These comparisons should be interpreted in the context of distinct methodological roles: co-folding models such as AlphaFold3 generate structural hypotheses, whereas P-RISE is designed to evaluate their energetic plausibility. Under the present benchmark conditions, this physics-based evaluation layer provides substantial added value for selecting near-native poses.

Our analysis suggests that the current limitations of AlphaFold3 in these RNA–ligand tasks may arise from at least three factors: the exploration bottleneck imposed by regression–based sampling, the failure of built–in confidence scores to reliably identify correct poses, and cascading errors originating from imperfect receptor structure prediction. Beyond this comparison, the systematic data experiments presented herein illustrate the difficulty faced by data-driven approaches in the data-poor regime. In the evaluated data-limited setting, both Pure-AI and Phys-AI models showed substantial variance across tasks, and none consistently exceeded the P-RISE reference. Phys-GPR retained partial pose-related information, but the physics-based scoring function more effectively preserved pose-level energetic contrast in the Dataset III benchmark. We further quantify that the physical prior embedded in P–RISE is information–theoretically equivalent to saving ~400–1 400 costly experimental measurements. Together, these results demonstrate that, in the absence of large datasets and in tasks requiring discrimination of subtle energetic differences, physically grounded modeling remains a reliable reference for tasks requiring subtle energetic discrimination under current RNA data conditions.

Our findings also point to a constructive direction for the next-generation co-folding models. Current architectures learn RNA–ligand complexes mainly from statistical/co-evolutionary and geometric signals. However, given the severe scarcity of ligand-bound RNA data, these signals alone may not be enough to distinguish subtle energetic differences. A promising and, in our view, viable direction is to make these models more energy aware. For example, we can incorporate explicit physics-based or energy-based objectives, scoring terms, or physically informed confidence estimation into training. In this way, structure generation and energetic discrimination can be optimized together rather than separately. In this sense, the physics-based evaluation layer shown here does not have to stay as an external post-hoc filter. It may eventually be integrated into data-driven models themselves. We see this as a natural convergence of physics-based and data-driven approaches for studying complex, ligand-bound RNA assemblies. We also acknowledge that RNA structure prediction is a rapidly evolving field, and the present comparison only reflects the state of these methods at the time of writing.

Finally, building on the present analyses, we outline an evolutionary roadmap for computational paradigms in RNA drug discovery and identify key short–term and long–term breakthrough directions. By explicitly identifying failure modes such as the “guanine challenge” and missing polarization effects, this study defines the capability limits of classical force–field–based approaches. This diagnosis immediately points to directions for the evolution of physical models themselves. For these challenging systems, near–term progress can be achieved through continued refinement of classical MD force field parameters and by introducing high–accuracy quantum-chemical calculations at critical interaction sites. More transformative advances may arise from the adoption of ML force fields (MLFFs) [69–71]. By learning from large–scale quantum–mechanical datasets, MLFFs offer the potential to overcome the traditional trade–off between accuracy and efficiency. With controlled computational cost, they may reproduce polarization effects and electronic density distributions at near–quantum accuracy, thereby addressing the intrinsic limitations of classical MM/GBSA in describing complex electronic phenomena.

However, the evolution of computational paradigms is unlikely to be a one–directional push on the physics front alone. It is more likely that substantial progress will stem from the systematic integration of physical models with data–driven learning. On this basis, we propose a heuristic three-stage framework describing the evolution of RNA drug discovery in three conceptual stages (Fig. 10).

**Figure 10.**
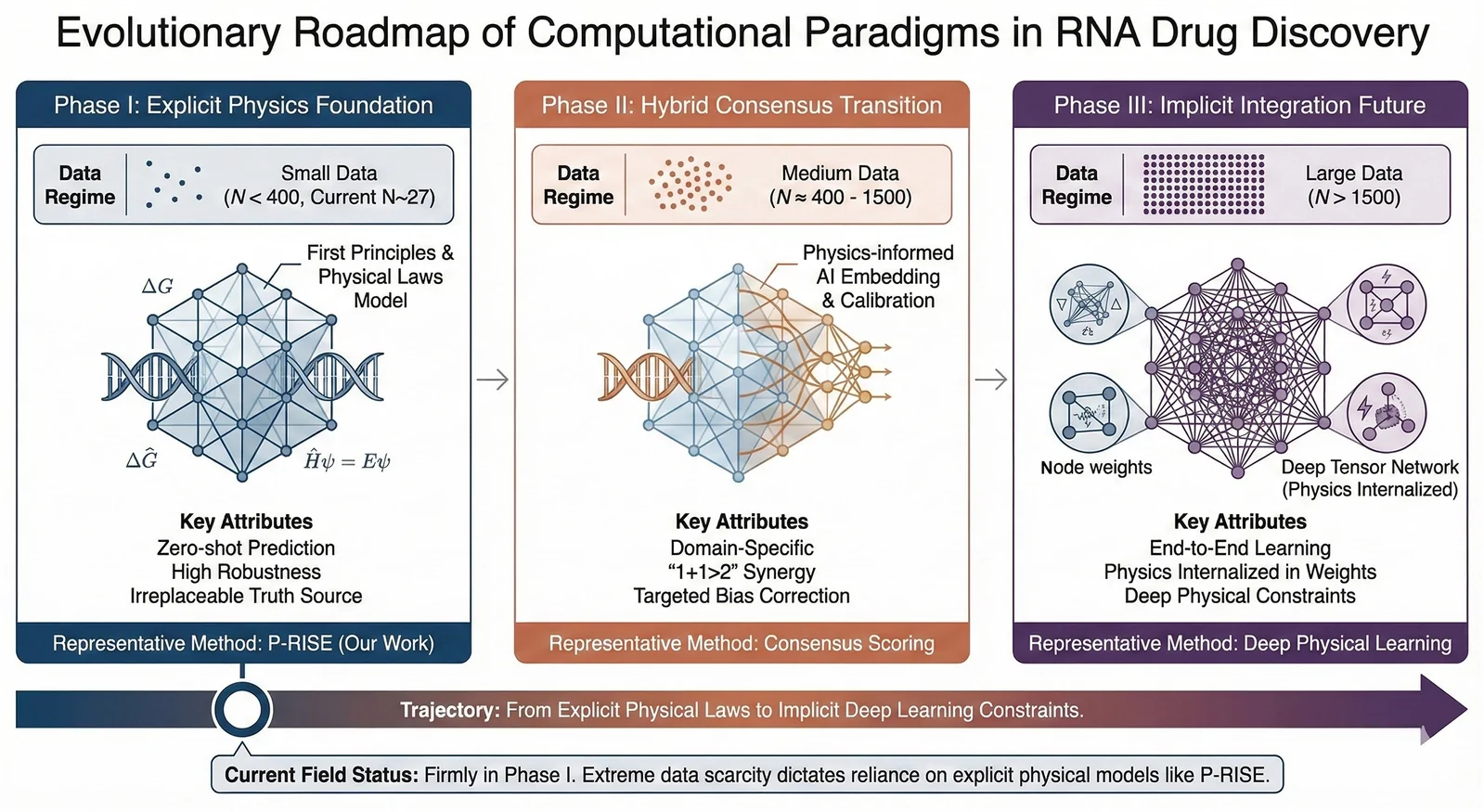
Conceptual framework for the evolving roles of explicit physics and data-driven models in RNA–ligand prediction. Based on the learning curve analysis, the optimal modeling strategy shifts as a function of sample size (*N*). Stage I (*N* <400): dominance of explicit physical models (P-RISE). In this regime, experimental data are insufficient to correct physical laws; thus, physics-based methods provide a robust baseline with meaningful conformational sensitivity. Stage II (*N* ~400–1500): the hybrid integration phase. ML algorithms benefit significantly from physical priors (Phys-AI), which serve to narrow the hypothesis space and enhance data efficiency. Stage III (*N* >1500): the era of implicit physical learning, where sufficient data allow “black-box” models to approximate the underlying physics.

Initially, in Stage4 I (“explicit physics foundation”) characterized by extremely small–sample regimes, physics–based methods such as P–RISE serve as foundational sources of prior knowledge, providing zero–shot predictive capability. As demonstrated herein, neither AnnapuRNA nor conventional ML models, including MLP and GPR, can overcome accuracy bottlenecks without sufficient data support. Under these conditions, direct application of a purely physics–based approach remains the most pragmatic strategy.

Subsequently, as dataset sizes grow to a moderate scale in Stage II (“hybrid integration”), the central strategy is expected to shift toward delta–learning. Here, P–RISE acts as the physical backbone, while data–driven components are trained specifically to learn the discrepancy between physical approximations and experimental ground truth, thereby correcting for specific blind spots such as polarization effects or base–specific biases.

Ultimately, with the potential emergence of large–scale datasets in Stage III (“implicit physical learning”), the long–term direction may involve internalizing physical constraints directly into neural network representations. In this regime, physics is no longer appended as an external module but could be embedded into model parameters, enabling end–to–end “deep physical” prediction.

Placing this roadmap in the context of our findings, it is crucial to recognize that the field currently resides in the early transition from Stage I to Stage II. While data remain scarce, the indiscriminate pursuit of increasingly complex AI architectures may not yield proportional gains. The importance of establishing P–RISE as a benchmark lies not only in its current performance but also in its role as a critical physical reference for future hybrid models.

Looking further ahead, as high–quality data accumulate and the field advances toward Stage III, the role of AI will undergo a qualitative shift. Future generative models should ideally move beyond predicting single static structures and aim to reconstruct entire binding free energy landscapes. In this vision, optimized MM/GBSA terms or even more accurate MLFF–derived energies could be directly incorporated as regularization components during training. Such integration holds the potential to transform AI from a black–box structure predictor into a genuine physical sampler that understands the relationship between energy and conformation. We believe that this closed–loop synergy between physics–enhanced AI and high–accuracy physical models will ultimately contribute to advancing RNA–ligand binding free energy calculations towards a new era of precision, reliability, and intelligence.

## Supplementary Material

gkag1062_Supplemental_File

## Data Availability

The curated datasets generated in this study are publicly available together with the P-RISE code, usage instructions, and tutorial materials at https://github.com/LiHui-CADD/P-RISE. The external datasets analyzed during the current study are available from the following sources: the PCCP dataset was obtained from the Supplementary Information of [24]; the FEP dataset was obtained from the Supplementary Information of  [27]; and the RNA–theophylline dataset is available at https://github.com/modernatx/rna_theophylline_free_energy_calculations.

## Code availability

The source code and data supporting this study are available at GitHub (https://github.com/LiHui-CADD/P-RISE) and have been archived in Zenodo (DOI: https://doi.org/10.5281/zenodo.22803435). The archived version corresponds to the code used in this paper.

## References

[1] Crick F . Central dogma of molecular biology. Nature. 1970;227:561–3. 10.1038/227561a0 4913914

[2] Tessaro F, Scapozza L. How ‘protein-docking’ translates into the new emerging field of docking small molecules to nucleic acids?. Molecules. 2020;25:2749. 10.3390/molecules25122749 PMC7355999 32545835

[3] Rizvi NF, Smith GF. RNA as a small molecule druggable target. Bioorg Med Chem Lett. 2017;27:5083–8. 10.1016/j.bmcl.2017.10.052 29097169

[4] Howe JA, Xiao L, Fischmann TO et al. Atomic resolution mechanistic studies of ribocil: a highly selective unnatural ligand mimic of the *E. coli* FMN riboswitch. RNA Biol. 2016;13:946–54. 10.1080/15476286.2016.1216304 PMC5056776 27485612

[5] Haack DB, Rudolfs B, Jin S et al. Scaffold-enabled high-resolution cryo-EM structure determination of RNA. Nat Commun. 2025;16:880. 10.1038/s41467-024-55699-5 PMC11751092 39837824

[6] Baird NJ, Inglese J, Ferre-D’Amare AR. Rapid RNA–ligand interaction analysis through high-information content conformational and stability landscapes. Nat Commun. 2015;6:8898. 10.1038/ncomms9898 PMC4686816 26638992

[7] Veenbaas SD, Felder S, Weeks KM. fpocketR: a platform for identification and analysis of ligand-binding pockets in RNA. ACS Chem Biol. 2026:21;151–9. 10.1021/acschembio.5c00805 PMC12904495 41403059

[8] Krishnan SR, Roy A, Wong L et al. DRLiPS: a novel method for prediction of druggable RNA–small molecule binding pockets using machine learning. Nucleic Acids Res. 2025;53: gkaf239. 10.1093/nar/gkaf239 PMC11963762 40173014

[9] Rekand IH, Brenk R. DrugPred_RNA-A tool for structure-based druggability predictions for RNA binding sites. J Chem Inf Model. 2021;61:4068–81. 10.1021/acs.jcim.1c00155 PMC8389535 34286972

[10] Wang J, Quan L, Jin Z et al. MultiModRLBP: a deep learning approach for multi-modal RNA–small molecule ligand binding sites prediction. IEEE J Biomed Health Inform. 2024;28:4995–5006. 10.1109/JBHI.2024.3400521 38739505

[11] Zhuo C, Zeng C, Yang R et al. RPflex: a coarse-grained network model for RNA pocket flexibility study. Int J Mol Sci. 2023;24:5497. 10.3390/ijms24065497 PMC10058308 36982570

[12] Szulc NA, Mackiewicz Z, Bujnicki JM et al. fingeRNAt-A novel tool for high-throughput analysis of nucleic acid–ligand interactions. PLoS Comput Biol. 2022;18:e1009783. 10.1371/journal.pcbi.1009783 PMC9197077 35653385

[13] Ma H, Gao L, Jin Y et al. RNA–ligand interaction scoring via data perturbation and augmentation modeling. Nat Comput Sci. 2025;5:648–60. 10.1038/s43588-025-00820-x 40555786

[14] Carvajal-Patino JG, Mallet V, Becerra D et al. RNAmigos2: accelerated structure-based RNA virtual screening with deep graph learning. Nat Commun. 2025;16:2799. 10.1038/s41467-025-57852-0 PMC11928640 40118849

[15] Abramson J, Adler J, Dunger J et al. Accurate structure prediction of biomolecular interactions with AlphaFold 3. Nature. 2024;630:493–500. 10.1038/s41586-024-07487-w PMC11168924 38718835

[16] Sun LZ, Jiang Y, Zhou Y et al. RLDOCK: a new method for predicting RNA–ligand interactions. J Chem Theory Comput. 2020;16:7173–83. 10.1021/acs.jctc.0c00798 PMC7882445 33095555

[17] Jiang Y, Chen SJ. RLDOCK method for predicting RNA–small molecule binding modes. Methods. 2022;197:97–105. 10.1016/j.ymeth.2021.01.009 PMC8333169 33549725

[18] Morley SD, Afshar M. Validation of an empirical RNA–ligand scoring function for fast flexible docking using Ribodock. J Comput Aided Mol Des. 2004;18:189–208. 10.1023/B:JCAM.0000035199.48747.1e 15368919

[19] Ruiz-Carmona S, Alvarez-Garcia D, Foloppe N et al. rDock: a fast, versatile and open source program for docking ligands to proteins and nucleic acids. PLoS Comput Biol. 2014;10:e1003571. 10.1371/journal.pcbi.1003571 PMC3983074 24722481

[20] Feng Y, Zhang K, Wu Q et al. NLDock: a fast nucleic acid–ligand docking algorithm for modeling RNA/DNA–ligand complexes. J Chem Inf Model. 2021;61:4771–82. 10.1021/acs.jcim.1c00341 34468128

[21] Korb O, Stützle T, Exner TE. PLANTS: application of ant colony optimization to structure-based drug design. Dorigo M Gambardella LM, Birattari M, Martinoli A, Poli R, Stützle T. (eds)In: *Ant Colony Optimization and Swarm Intelligence. ANTS 2006. Lecture Notes in Computer Science, vol 4150*. Berlin Heidelberg: Springer, 2006;247–58. 10.1007/11839088 10.1007/11839088_22

[22] Tan LH, Kwoh CK, Mu Y. RmsdXNA: RMSD prediction of nucleic acid–ligand docking poses using machine-learning method. Brief Bioinform. 2024;25:bbae166. 10.1093/bib/bbae166 PMC11063749 38695120

[23] Stefaniak F, Bujnicki JM. AnnapuRNA: a scoring function for predicting RNA–small molecule binding poses. PLoS Comput Biol. 2021;17:e1008309. 10.1371/journal.pcbi.1008309 PMC7877745 33524009

[24] Jiang D, Du H, Zhao H et al. Assessing the performance of MM/PBSA and MM/GBSA methods. 10. Prediction reliability of binding affinities and binding poses for RNA-ligand complexes. Phys Chem Chem Phys. 2024;26:10323–35. 10.1039/D3CP04366E 38501198

[25] Fu H, Chen H, Cai W et al. BFEE2: automated, streamlined, and accurate absolute binding free-energy calculations. J Chem Inf Model. 2021;61:2116–23. 10.1021/acs.jcim.1c00269 33906354

[26] Rasouli A, Pickard FC 4th, Sur S et al. Essential considerations for free energy calculations of RNA–small molecule complexes: lessons from the theophylline-binding RNA aptamer. J Chem Inf Model. 2025;65:223–39. 10.1021/acs.jcim.4c01505 PMC11734693 39699235

[27] Abramyan AM, Bochicchio A, Wu C et al. Accurate physics-based prediction of binding affinities of RNA- and DNA-targeting ligands. J Chem Inf Model. 2025;65:1392–403. 10.1021/acs.jcim.4c01708 39883536

[28] Bonilla SL, Jang K. Challenges, advances, and opportunities in RNA structural biology by Cryo-EM. Curr Opin Struct Biol. 2024;88:102894. 10.1016/j.sbi.2024.102894 39121532

[29] Pallikkavaliyaveetil N, Chandrasekaran S. Small data, big challenges: machine- and deep-learning strategies for data-limited drug discovery. Adv Drug Deliv Rev. 2026;229:115762. 10.1016/j.addr.2025.115762 PMC13094559 41421504

[30] Rahnenfuhrer J, De Bin R, Benner A et al. Statistical analysis of high-dimensional biomedical data: a gentle introduction to analytical goals, common approaches and challenges. BMC Med. 2023;21:182. 10.1186/s12916-023-02858-y PMC10186672 37189125

[31] Jiang J, Zhang CH, Ke L et al. A review of machine learning methods for imbalanced data challenges in chemistry. Chem Sci. 2025;16:7637–58. 10.1039/D5SC00270B PMC12013631 40271022

[32] Cheng T, Li X, Li Y et al. Comparative assessment of scoring functions on a diverse test set. J Chem Inf Model. 2009;49:1079–93. 10.1021/ci9000053 19358517

[33] Li Y, Su M, Liu Z et al. Assessing protein–ligand interaction scoring functions with the CASF-2013 benchmark. Nat Protoc. 2018;13:666–80. 10.1038/nprot.2017.114 29517771

[34] Su M, Yang Q, Du Y et al. Comparative assessment of scoring functions: the CASF-2016 update. J Chem Inf Model. 2019;59:895–913. 10.1021/acs.jcim.8b00545 30481020

[35] Jiang DJ, Zhao HF, Du HY et al. How good are current docking programs at nucleic acid–ligand docking? A comprehensive evaluation. J Chem Theory Comput. 2023;19:5633–47. 10.1021/acs.jctc.3c00507 37480347

[36] Zhang C, Shine M, Pyle AM et al. US-align: universal structure alignments of proteins, nucleic acids, and macromolecular complexes. Nat Methods. 2022;19:1109–15. 10.1038/s41592-022-01585-1 36038728

[37] Lu C, Wu C, Ghoreishi D et al. OPLS4: improving force field accuracy on challenging regimes of chemical space. J Chem Theory Comput. 2021; 17, 17,4291–300. 10.1021/acs.jctc.1c00302 34096718

[38] Gong Z, Xiao Y, Xiao Y. RNA stability under different combinations of amber force fields and solvation models. J Biomol Struct Dyn. 2010;28:431–41. 10.1080/07391102.2010.10507372 20919758

[39] Zhao J, Kennedy SD, Turner DH. Nuclear magnetic resonance spectra and AMBER OL3 and ROC-RNA simulations of UCUCGU reveal force field strengths and weaknesses for single-stranded RNA. J Chem Theory Comput. 2022;18:1241–54. 10.1021/acs.jctc.1c00643 34990548

[40] Zgarbova M, Otyepka M, Sponer J et al. Refinement of the Cornell et al. nucleic acids force field based on reference quantum chemical calculations of glycosidic torsion profiles. J Chem Theory Comput. 2011; 7, 2886–902. 10.1021/ct200162x PMC3171997 21921995

[41] Yildirim I, Stern HA, Tubbs JD et al. Benchmarking AMBER force fields for RNA: comparisons to NMR spectra for single-stranded r(GACC) are improved by revised χ torsions. J Phys Chem B. 2011;115:9261–70. 10.1021/jp2016006 PMC3140773 21721539

[42] Wang J, Wolf RM, Caldwell JW et al. Development and testing of a general amber force field. J Comput Chem. 2004;25:1157–74. 10.1002/jcc.20035 15116359

[43] Sousa da Silva AW, Vranken WF. ACPYPE–AnteChamber PYthon Parser interfacE. BMC Res Notes. 2012;5:367. 10.1186/1756-0500-5-367 PMC3461484 22824207

[44] Jorgensen WL, Chandrasekhar J, Madura JD et al. Comparison of simple potential functions for simulating liquid water. J Chem Phys. 1983;79:926–35. 10.1063/1.445869

[45] Gotz AW, Williamson MJ, Xu D et al. Routine microsecond molecular dynamics simulations with AMBER on GPUs. 1. Generalized Born. J Chem Theory Comput. 2012;8:1542–55. 10.1021/ct200909j PMC3348677 22582031

[46] Salomon-Ferrer R, Gotz AW, Poole D et al. Routine microsecond molecular dynamics simulations with AMBER on GPUs. 2. Explicit solvent particle mesh Ewald. J Chem Theory Comput. 2013;9:3878–88. 10.1021/ct400314y 26592383

[47] Shilov IY, Lyashchenko AK. Comment on “Predicting activity coefficients with the Debye–Huckel theory using concentration dependent static permittivity”. Aiche J. 2022;68:1.

[48] Hasted JB, Ritson DM, Collie CH. Dielectric properties of aqueous ionic solutions. Parts I and II. J Chem Phys. 1948;16:1–21. 10.1063/1.1746645

[49] Lawson CL, Berman HM, Chen L et al. The Nucleic Acid Knowledgebase: a new portal for 3D structural information about nucleic acids. Nucleic Acids Res. 2023;52:D245–54. 10.1093/nar/gkad957 PMC10767938 37953312

[50] Roy D, Kovalenko A. Biomolecular simulations with the three-dimensional reference interaction site model with the Kovalenko–Hirata closure molecular solvation theory. Int J Mol Sci. 2021;22:5061. 10.3390/ijms22105061 PMC8151972 34064655

[51] Case DA, Aktulga HM, Belfon K et al. ambertools. j Chem Inf Model. 2023;63:6183–91. 10.1021/acs.jcim.3c01153 PMC10598796 37805934

[52] Tosco P, Stiefl N, Landrum G. Bringing the MMFF force field to the RDKit: implementation and validation. J Cheminform. 2014;6: 37. 10.1186/s13321-014-0037-3

[53] Cock PJA, Antao T, Chang JT et al. Biopython: freely available Python tools for computational molecular biology and bioinformatics. Bioinformatics. 2009;25:1422–3. 10.1093/bioinformatics/btp163 PMC2682512 19304878

[54] Vilar S, Cozza G, Moro S. Medicinal chemistry and the molecular operating environment (MOE): application of QSAR and molecular docking to drug discovery. CTMC. 2008;8:1555–72. 10.2174/156802608786786624 19075767

[55] Lorenz R, Bernhart SH, Siederdissen CHZ et al. ViennaRNA package 2.0. Algorithms Mol Biol. 2011;6:26. 10.1186/1748-7188-6-26 PMC3319429 22115189

[56] Chen T, Guestrin C. 2016; XGBoost: a scalable tree boosting system. Proceedings of the 22nd ACM SIGKDD International Conference on Knowledge Discovery and Data Mining. 10.1145/2939672.2939785

[57] Pedregosa F, Varoquaux G, Gramfort A et al. Scikit-learn: machine learning in Python. J Mach Learn Res. 2011;12:2825–30.

[58] Mohr F, van Rijn JN. Learning curves for decision making in supervised machine learning: a survey. Mach Learn. 2024;113:8371–425. 10.1007/s10994-024-06619-7

[59] Lundberg SM, Erion G, Chen H et al. From local explanations to global understanding with explainable AI for trees. Nat Mach Intell. 2020;2:56–67. 10.1038/s42256-019-0138-9 PMC7326367 32607472

[60] Bottaro S, Bussi G, Pinamonti G et al. Barnaba: software for analysis of nucleic acid structures and trajectories. RNA. 2019;25:219–31. 10.1261/rna.067678.118 PMC6348988 30420522

[61] Bottaro S, Di Palma F, Bussi G. The role of nucleobase interactions in RNA structure and dynamics. Nucleic Acids Res. 2014;42:13306–14. 10.1093/nar/gku972 PMC4245972 25355509

[62] Fischer NM, Poleto MD, Steuer J et al. Influence of Na^+^ and Mg^2+^ ions on RNA structures studied with molecular dynamics simulations. Nucleic Acids Res. 2018;46:4872–82. 10.1093/nar/gky221 PMC6007214 29718375

[63] Nguyen HT, Thirumalai D. Charge density of cation determines inner versus outer shell coordination to phosphate in RNA. J Phys Chem B. 2020;124:4114–22. 10.1021/acs.jpcb.0c02371 32342689

[64] Fedotova MV, Dmitrieva OA. Ion-selective interactions of biologically relevant inorganic ions with alanine zwitterion: a 3D-RISM study. Amino Acids. 2015;47:1015–23. 10.1007/s00726-015-1930-4 25663287

[65] Srivastava R, Chattopadhyaya M, Bandyopadhyay P. Calculation of salt-dependent free energy of binding of beta-lactoglobulin homodimer formation and mechanism of dimer formation using molecular dynamics simulation and three-dimensional reference interaction site model (3D-RISM): diffuse salt ions and non-polar interactions between the monomers favor the dimer formation. Phys Chem Chem Phys. 2020;22:2142–56. 10.1039/c9cp05578a 31912070

[66] Sun LZ, Zhang JX, Chen SJ. MCTBI: a web server for predicting metal ion effects in RNA structures. RNA. 2017;23:1155–65. 10.1261/rna.060947.117 PMC5513060 28450533

[67] Olenginski LT, Wierzba AJ, Laursen SP et al. Designing small molecules targeting a cryptic RNA binding site through base displacement. Nat Chem Biol. 2026;22:1004–1013. 10.1038/s41589-025-02018-8 PMC12688817 40883492

[68] Masters MR, Mahmoud AH, Lill MA. Investigating whether deep learning models for co-folding learn the physics of protein–ligand interactions. Nat Commun. 2025;16:8854. 10.1038/s41467-025-63947-5 PMC12501370 41053181

[69] Gao X, Ramezanghorbani F, Isayev O et al. TorchANI: a free and open source PyTorch-based deep learning implementation of the ANI neural network potentials. J Chem Inf Model. 2020;60:3408–15. 10.1021/acs.jcim.0c00451 32568524

[70] Pickering I, Xue JZ, Huddleston K et al. TorchANI 2.0: an extensible, high-performance library for the design, training, and use of NN-IPs. J Chem Inf Model. 2025;65:11656–71. 10.1021/acs.jcim.5c01853 41105703

[71] Pickering I, Semelak JA, Xue JZ et al. TorchANI-Amber: bridging neural network potentials and classical biomolecular simulations. J Phys Chem B. 2025;129:11927–38. 10.1021/acs.jpcb.5c05725 41213858

