# Supplementary material for: Physical determinants of reliable RNA–ligand binding prediction revealed by P-RISE
Source: Nucleic Acids Res. 2026 Nov 2;54(20):gkag1062. doi: 10.1093/nar/gkag1062 (PMC13725139; doi:10.1093/nar/gkag1062)
Supplement: Supplementary file 1 [file gkag1062_supplemental_file.docx]

### Supporting Information for “Physical determinants of reliable RNA-ligand binding prediction revealed by P-RISE”

Hui Li^1^, Youchao Deng^1^, Zhuoxi Jiang^1^, Xinyu Gu^1,*^, Dejun Jiang^1,*^, Dongsheng Cao^1,*^

^1^Xiangya School of Pharmaceutical Sciences, Central South University, Changsha 410004, China.

*Corresponding authors

Xinyu Gu

Email: xgu123@csu.edu.cn

Dejun Jiang

Email: jiang_dj@zju.edu.cn

Dongsheng Cao

Email: oriental-cds@163.com

**Supplementary Figures:**

**Figure S1.** Asymmetric dual ligands in excluded systems (PDB ID: 4LVW, 4LVY).

**Figure S2.** Violin plots of conformation distribution for dataset III, showing RMSD distributions for 20 PLANTS poses per system. The "Success cutoff" indicates the poses that are considered successful, and the percentage of such poses in the system is shown below the PDB ID number on the horizontal axis.

**Figure S3.** Overview of Categorized dataset I. The dataset is diverse in terms of energy decomposition, physiological function and charge. (A) Distribution of energy-decomposition classes. (B) Distribution of physiological functions. (C) Distribution of ligand net charges. (D) Distribution of RNA net charges.

**Figure S4.** Detailed performance (Rₚ) of Ion Concentration and Type. Pearson correlation (R_p_) between predicted and experimental binding free energies is shown versus salt concentration for KCl and MgCl_2_ using GBSA (GB) and PBSA (PB) models. (A, B) OL3 force field. (C, D) YIL force field, minimized structures. (E, F) YIL force field, 1-2 ns MD snapshots.

**Figure S5.** Heatmap of Rₚ vs. Ion Concentration for PBSA models. (A) OL3-PBSA. (B) YIL-PBSA. Rows indicate salt type (NaCl, KCl, MgCl_2_) and columns indicate salt concentration (0.05, 0.15, 0.5, 1 and 2 M). Colour represents the Pearson correlation (R_p_).

**Figure S6.** RMSD trajectories for all 27 systems in 0.5 M MgCl₂.

**Figure S7.** RMSD trajectories for all 27 systems in 2M NaCl.

**Figure S8.** RMSD trajectories for all 27 systems in 1M KCl.

**Figure S9.** Temporal stability of the entropy correction and statistical robustness of the Dataset I ranking. All panels use the P-RISE protocol (OL3 force field, 0.5 M MgCl₂, ε_in_ = 20, ε_out_ = fix) on the 27 complexes of Dataset I; time windows are labelled 0-2, 2-4, 4-6, 6-8 and 8-10 ns. (A) Per-window QH entropy correction -TΔS for each system, expressed relative to its own five-window mean. Thin grey lines, individual complexes; bold black line, across-system mean; shaded band, ±1 SD. (B) Between-system standard deviation of -TΔS versus window, remaining within 0.64-0.75 kcal/mol across the full trajectory. (C) Per-window Pearson correlation R_p_ between predicted and experimental ΔG, near-identical for the 1-2 and 4-6 ns windows and declining beyond 6 ns. (D) Reproducibility of -TΔS across three independent random seeds; systems ordered by residue count, 4–6 ns -TΔS on the y-axis, symbols and error bars give per-system mean ± SD over seeds. The mean per-system SD across seeds is 0.82 kcal/mol (≈17% of the 7.9-27.0 kcal/mol between-system range). The two largest complexes, 4LVZ and 4LW0, show the lowest -TΔS with very small dispersion, reflecting entropy loss concentrated in a small number of local degrees of freedom rather than a computational artefact. (E) The most demanding case, the 90-residue complex 4LW0, across all five windows and three replicates; window-to-window SD = 0.06 and seed-to-seed SD = 0.07 kcal/mol (<2% of the between-system range). (F) Predicted versus experimental ΔG for the full Dataset I from the seed-averaged predictions (R_p_ = 0.631); error bars are per-complex SD across seeds. The three seeds individually give R_p_ = 0.62 ± 0.07, and the single-run reference value (0.696) lies within one SD of this mean. Predicted values are systematically more negative than experiment, a known bias of endpoint methods; because only the ranking is used, a best-fit line with its 95% confidence band is shown rather than the y = x diagonal. The largest seed-to-seed dispersion is that of 2LWK, the conformationally labile, marginally bound complex already identified in the Figure 3 analysis. (G) Leave-one-out analysis: R_p_ of the remaining 26 systems when each complex is removed in turn (seed-averaged predictions; full-set R_p_ = 0.631, red line). Values span 0.577–0.670, confirming that no single complex drives the overall correlation.

**Figure S10.** Detailed interaction site map of the four nucleotides in RNA.

**Figure S11.** Ensemble-aware control benchmark based on 41 NMR-derived RNA-ligand complexes. (A) Top-N success rate, success defined as at least one pose with ligand RMSD < 3 Å among the top N. Blue solid line, P-RISE on the NMR control set under the best-match criterion (Top-1 61.0%, Top-3 80.5%); orange dashed line, the identical ranking evaluated under the ensemble-average criterion (Top-1 43.9%); purple solid line, PLANTS scoring baseline (Top-1 36.6%, Top-3 68.3%); faint dotted lines, P-RISE and PLANTS on crystallographic Dataset III, shown as a trend reference only. For the decoy composition of this control set the random expectations at Top-1 and Top-3 are 26.5% and 60.3%. The two sets are not directly comparable in absolute terms, differing in the number of systems (41 vs 20) and in the origin of the receptor conformations (ensemble docking vs a single crystallographic receptor); only the improvement over the respective baselines is compared, namely +24.4 and +30.0 percentage points at Top-1, of the same order. (B) Rationale for the choice of criterion. The ordinate is the median reference-state penalty, RMSD_avg - RMSD_best, of the Top-1 pose. The two criteria differ by 17.1 percentage points in Top-1 success rate, and this difference is almost perfectly linear in ensemble divergence (R_p_ = 0.97; Spearman ρ = 0.96), showing that it originates from the construction of the reference-state geometry rather than from discriminative power: a pose that exactly reproduces one genuine conformer of the ensemble is still systematically penalised by its average deviation from the remaining, more distant conformers. The best-match criterion is therefore used in the remainder of the figure; ensemble-average results are reported alongside for transparency, and their lower values should not be interpreted as reduced discriminative capability. (C) Per-system decoy distribution and P-RISE Top-1 selection. Systems are ordered by the best-match RMSD of their Top-1 pose. Light-blue violins with grey points show the RMSD distribution of all poses of each system; every system spans 0-10 Å and contains at least one pose below 3 Å, so any failure is a ranking rather than a sampling-coverage problem. Red circles, P-RISE Top-1; where Top-1 is not a hit, a blue circle marks the highest-ranked near-native pose and its rank is annotated above (gold, rank 2-5; red, rank > 5). The dashed red line is the 3 Å success threshold. Top-1 is successful for 25/41 systems (61.0%); the first near-native pose lies within the top 3 for 33 and within the top 5 for 35 systems. Systems in which ranking fails have significantly more solvent-exposed binding sites than the remainder, consistent with the known limitation of implicit-solvent models at highly exposed, desolvation-dominated sites. (D) Self-consistency of the pooled benchmark. The ordinate is the best-match RMSD of the P-RISE Top-1 pose; the dashed red line is the 3 Å threshold. The two quantities are uncorrelated (R_p_ = 0.14; Spearman ρ = 0.15), indicating that pooled evaluation introduces no systematic bias coupled to conformational heterogeneity and that evaluation error does not grow with system flexibility. Colours in (B) and (D) are consistent: blue, Top-1 hit; grey, first near-native pose ranked 2-5; red, ranked beyond 5.

**Figure S12.** Systematic hyperparameter optimization landscapes comparing baseline and enhanced feature integrations. Parallel coordinate plots visualize the rigorous hyperparameter search space across five machine learning algorithms. The figure is arranged into two columns to demonstrate the impact of feature augmentation: the left column displays optimization trajectories for models trained exclusively on physicochemical descriptors (static PDB-based); the right column presents models trained on a comprehensive feature set integrating physicochemical descriptors with P-RISE energy and MD dynamics terms. The panels are organized by algorithm (from top to bottom): XGBoost, Random Forest (RF), Support Vector Machine (SVM), Gaussian Process Regression (GPR), and Multilayer Perceptron (MLP). In each plot, vertical axes represent the specific hyperparameters tuned. Individual flowing lines depict distinct hyperparameter configuration trials, colored according to the validation Pearson correlation coefficient (Rp) on a unified scale to facilitate direct performance comparison. The prominent red trajectory identifies the global optimal configuration identified for each model. The high density of background curves illustrates the exhaustive coverage of the search space, ensuring the robustness of the optimized models.

**Supplementary Tables:**

**Table S1.** Overview of the 27 RNA-Ligand complexes in the benchmark dataset I for binding affinity.

| PDB ID | K_i_ / K_d_ (μM) | ΔG_exp_ (kcal/mol) |
| --- | --- | --- |
| 3NPN | 0.032 | -10.16 |
| 1UTS | 0.039 | -10.05 |
| 2AU4 | 0.075 | -9.65 |
| 2BE0 | 0.13 | -9.33 |
| 2N0J | 0.33 | -8.78 |
| 4LVZ | 0.5 | -8.54 |
| 1Q8N | 0.8 | -8.25 |
| 2KGP | 1 | -8.13 |
| 3S4P | 1 | -8.12 |
| 1UUD | 1.54 | -7.87 |
| 1FYP | 2.5 | -7.85 |
| 1BYJ | 2 | -7.71 |
| 2F4T | 2.2 | -7.65 |
| 2KU0 | 2.6 | -7.56 |
| 2O3W | 2.6 | -7.55 |
| 6HAG | 3.7 | -7.35 |
| 2MXS | 5.1 | -7.16 |
| 1QD3 | 5.9 | -7.07 |
| 2F4U | 5.9 | -7.07 |
| 2KTZ | 9 | -7.04 |
| 2KX8 | 6.31 | -7.03 |
| 4LW0 | 8 | -6.89 |
| 2F4S | 19 | -6.38 |
| 2O3X | 50 | -5.8 |
| 2LWK | 50.5 | -5.79 |
| 2L94 | 360 | -4.64 |
| 1ARJ | 1000 | -4.03 |

**Table S2.** Overview of categorized benchmark dataset I.

| System | Energy Decomposition | Ligand Charge | RNA Charge | Function |
| --- | --- | --- | --- | --- |
| 2KU0 | G | 3 | -34 | Transcription Regulating |
| 3NPN | A | 2 | -51 | Riboswitch |
| 1BYJ | G | 5 | -21 | Protein Synthesis |
| 2MXS | A | 5 | -21 | Riboswitch |
| 2O3W | G | 5 | -39 | Protein Synthesis |
| 2L94 | U | 4 | -40 | Transcription Regulating |
| 2AU4 | G | 4 | -36 | Other |
| 2O3X | G | 4 | -40 | Protein Synthesis |
| 2F4T | G | 5 | -37 | Protein Synthesis |
| 1Q8N | G | 1 | -36 | Other |
| 2KTZ | G | 3 | -34 | Transcription Regulating |
| 1ARJ | A | 3 | -25 | Transcription Regulating |
| 1UUD | U | 3 | -25 | Transcription Regulating |
| 2F4U | G | 5 | -35 | Protein Synthesis |
| 2N0J | A | 4 | -22 | Riboswitch |
| 4LVZ | A | 0 | -88 | Riboswitch |
| 2KGP | G | 2 | -22 | Other |
| 2BE0 | G | 6 | -30 | Protein Synthesis |
| 1QD3 | G | 6 | -22 | Transcription Regulating |
| 4LW0 | A | 0 | -88 | Riboswitch |
| 1FYP | U | 5 | -21 | Protein Synthesis |
| 3S4P | G | 6 | -28 | Protein Synthesis |
| 2KX8 | U | 2 | -39 | Transcription Regulating |
| 2LWK | U | 1 | -30 | Other |
| 6HAG | C | 2 | -40 | Riboswitch |
| 1UTS | U | 3 | -25 | Transcription Regulating |
| 2F4S | G | 4 | -36 | Protein Synthesis |

**Table S3.** Details of the Benzimidazole system in benchmark dataset II.

| Ligand | Energy Decomposition | Ligand Charge | RNA Charge | ΔG_exp_ (kcal/mol) |
| --- | --- | --- | --- | --- |
| ligand13_p_1 | A | 3 | -32 | -8.4 |
| ligand_11_n_2 | A | 3 | -32 | -7.9 |
| ligand_10_n | G | 3 | -32 | -7.4 |
| ligand_4_2 | A | 3 | -32 | -7 |
| ligand_5_2 | A | 3 | -32 | -7 |
| ligand_3_n_2 | A | 3 | -32 | -6.5 |
| ligand_2f_1 | A | 2 | -32 | -6 |
| ligand_2i_1 | A | 2 | -32 | -5.9 |
| ligand_1_2 | A | 2 | -32 | -5.5 |
| ligand_2j_2 | A | 2 | -32 | -5.5 |
| ligand_2b_2 | A | 2 | -32 | -5.3 |
| ligand_2c_2 | A | 2 | -32 | -5.3 |
| ligand_9_p_2 | G | 3 | -32 | -5 |

**Table S4.** Details of the Rbt system in benchmark dataset II.

| Ligand | Energy Decomposition | Ligand Charge | RNA Charge | ΔG_exp_ (kcal/mol) |
| --- | --- | --- | --- | --- |
| 203_h | U | 3 | -26 | -7.9 |
| 161_h | A | 3 | -26 | -7.6 |
| 202_h | A | 3 | -26 | -7.5 |
| 202 | U | 2 | -26 | -7.5 |
| 392_h | A | 3 | -26 | -7.5 |
| 160_h | A | 3 | -26 | -7.2 |
| 394 | A | 2 | -26 | -6.6 |
| 442 | A | 2 | -26 | -6.1 |
| 402 | A | 2 | -26 | -5.5 |
| 393 | A | 2 | -26 | -4.8 |

**Table S5.** Details of the Flavins system in benchmark dataset II.

| Ligand | Energy Decomposition | Ligand Charge | RNA Charge | ΔG_exp_ (kcal/mol) |
| --- | --- | --- | --- | --- |
| BRX1224_1 | A | 0 | -109 | -11.3 |
| 6dn1_natural | A | -1 | -109 | -11 |
| 6dn2_natural | A | 0 | -109 | -10.6 |
| BRX1555-6DN3_natural | A | 0 | -109 | -10.1 |
| BRX1072 | A | 0 | -109 | -9.8 |
| BRX1675 | G | 0 | -109 | -9.4 |
| BRX931_1 | U | -1 | -109 | -9.4 |
| BRX857 | A | -1 | -109 | -9.1 |
| BRX897 | A | -1 | -109 | -8.6 |
| BRX247 | U | 0 | -109 | -7.2 |

**Table S6. Paired ΔR_p_ comparisons quantifying the statistical robustness of each P-RISE design choice on Dataset I (N = 27).**

| Determinant | Retained setting vs control | Paired ΔR_p_ | 95% CI | Statistical resolution |
| --- | --- | --- | --- | --- |
| Internal dielectric | ε_in_ = 20 vs ε_in_ = 1 | +0.493 | [0.085, 0.884] | resolved |
| Ionic strength | 0.5 M MgCl₂ vs no added salt | +0.390 | [−0.050, 0.594] | large but unresolved |
| Production window | 4–6 ns vs 8–10 ns | +0.185 | [0.063, 0.304] | resolved |
| Conformational entropy | + QH correction vs no entropy term | +0.074 | [−0.049, 0.216] | positive, unresolved |
| External dielectric | ε_out_ = fix vs ε_out_ = 80 | +0.005 | [−0.004, 0.013] | null effect |

Paired comparisons across all N = 27 complexes of Dataset I; ΔR_p_ is the change in Pearson correlation on switching from the control setting to the setting retained in P-RISE, computed on the same systems so that system-specific noise cancels. Confidence intervals are bootstrap intervals over 10 000 resamples of the 27 paired differences. Intervals excluding zero are described as resolved at the present sample size; the remaining entries are reported with their point estimates and intervals unchanged, and are discussed in the text as effects that are directionally consistent but not individually resolved at N = 27.

**Table S7.** Details of systems for pose prediction in benchmark dataset III.

| PDB ID | Experimental Method | Number of submitted isomers in PDB dataset | Resolution (Å) | Number of ligands in RNA pocket |
| --- | --- | --- | --- | --- |
| 2F4U | X-RAY | 1 | 2.6 | 1 |
| 2O3X | X-RAY | 1 | 2.9 | 1 |
| 3Q50 | X-RAY | 1 | 2.75 | 1 |
| 4NFO | X-RAY | 1 | 1.96 | 1 |
| 6U8F | X-RAY | 1 | 2.81 | 1 |
| 2OE8 | X-RAY | 1 | 1.8 | 1 |
| 5KVJ | X-RAY | 1 | 2.26 | 1 |
| 2ET8 | X-RAY | 1 | 2.5 | 1 |
| 4ERJ | X-RAY | 1 | 3 | 1 |
| 5SWE | X-RAY | 1 | 3 | 1 |
| 2F4S | X-RAY | 1 | 2.8 | 1 |
| 2O3V | X-RAY | 1 | 2.8 | 1 |
| 4LVZ | X-RAY | 1 | 1.77 | 1 |
| 5VCF | X-RAY | 1 | 2.8 | 1 |
| 2F4T | X-RAY | 1 | 3 | 1 |
| 2O3W | X-RAY | 1 | 2.8 | 1 |
| 3NPN | X-RAY | 1 | 2.79 | 1 |
| 4LW0 | X-RAY | 1 | 1.89 | 1 |
| 5VCI | X-RAY | 1 | 2.6 | 1 |
| 6JBF | X-RAY | 1 | 2.6 | 1 |
